# Protection studies of an excretory–secretory protein HcABHD against *Haemonchus contortus* infection

**DOI:** 10.1186/s13567-020-00871-0

**Published:** 2021-01-06

**Authors:** Mingmin Lu, Xiaowei Tian, Yang Zhang, Wenjuan Wang, Ai-Ling Tian, Kalibixiati Aimulajiang, Lianrui Liu, Charles Li, Ruofeng Yan, Lixin Xu, Xiaokai Song, Xiangrui Li

**Affiliations:** 1 MOE Joint International Research Laboratory of Animal Health and Food Safety, College of Veterinary Medicine, Nanjing Agricultural University, Nanjing, 210095 Jiangsu P. R. China; 2 State Key Laboratory of Veterinary Etiological Biology, Lanzhou Veterinary Research Institute, Chinese Academy of Agricultural Sciences, Lanzhou, 730046 Gansu P. R. China; 3 Animal Biosciences and Biotechnology Laboratory, Beltsville Agricultural Research Center, Agricultural Research Service, U.S. Department of Agriculture, Beltsville, MD 20705 USA

**Keywords:** *H. contortus*, excretory–secretory protein, Α/β-hydrolase, immunization, goats

## Abstract

Unlike the successful immunization of native *H. contortus* antigens that contributed to the realization of the first commercial vaccine Barbervax, not many studies revealed the encouraging protective efficacies of recombinant *H. contortus* antigens in laboratory trials or under field conditions. In our preliminary study, *H. contortus* α/β-hydrolase domain protein (HcABHD) was demonstrated to be an immunomodulatory excretory–secretory (ES) protein that interacts with goat T cells. We herein evaluated the protective capacities of two HcABHD preparations, recombinant HcABHD (rHcABHD) antigen and anti-rHcABHD IgG, against *H. contortus* challenge via active and passive immunization trials, respectively. Parasitological parameter, antibody responses, hematological pathology and cytokine profiling in unchallenged and challenged goats were monitored and determined throughout both trials. Subcutaneous administration of rHcABHD with Freund adjuvants elicited protective immune responses in challenged goats, diminishing cumulative fecal egg counts (FEC) and total worm burden by 54.0% and 74.2%, respectively, whereas passive immunization with anti-rHcABHD IgG conferred substantial protection to challenged goats by generating a 51.5% reduction of cumulative FEC and a 73.8% reduction of total worm burden. Additionally, comparable changes of mucosal IgA levels, circulating IgG levels, hemoglobin levels, and serum interleukin (IL)-4 and IL-17A levels were observed in rHcABHD protein/anti-rHcABHD IgG immunized goats in both trials. Taken together, the recombinant version of HcABHD might have further application under field conditions in protecting goats against *H. contortus* infection, and the integrated immunological pipeline of ES antigen identification, screening and characterization may provide new clues for further development of recombinant subunit vaccines to control *H. contortus*.

## Introduction

*Haemonchus contortus* is a highly pathogenic gastrointestinal nematode with a developmental life cycle including three free-living larval stages and two parasitic stages. This parasitic nematode resides in the abomasum of ruminants, particularly in sheep and goats, and causes anaemia, haemorrhagic gastritis and relevant complications [1]. Given its poor productivity and widespread occurrence, haemonchosis has resulted in substantial economic losses and is designated as one of the salient constraints on the livestock industry worldwide, especially in tropical and subtropical regions. Currently, chemical strategies using active anthelmintic groups like benzimidazoles, imidazothiazoles, tetrahydropyrimidines, salicylanilides, macrocyclic lactones and amino-acetonitrile derivatives remain the mainstay for the treatment or prevention of haemonchosis [2]. However, with the occurrence of global anthelmintic-resistance, alternative nonchemical strategies are imperative to be developed and employed for the increasing demands of drug-free animal production [3].

Alongside grazing and nutritional management, underpinning the immunoprophylactic control of *H. contortus* via vaccination has been a long-term goal of many parasitologists during the last 20 years [4]. Significant efforts have been made to identify the key antigens as vaccine candidates from the developmental life-cycle stages of *H. contortus* via integrated immunoproteomic and immunogenomic approaches, e.g. H-gal-GP [5], H11 [6], GA1 [7], Hc-sL3 [8]. In 2014, the first commercially available vaccine Barbervax encompassing enriched native gut-derived antigens H-gal-GP and H11 was authorized in Australia and produced at an industrial scale via *H. contortus* harvested from donor sheep based on processing and production technology [4]. As Barbervax is made up of native hidden antigens that rely on frequent boosting to generate high levels of circulating antibodies, the development of alternative vaccines like recombinant subunit vaccines still needs to be further investigated.

Parasitic helminths could release excretory–secretory (ES) products into the host environment actively or passively to ensure their survival [9]. Investigations of these ES proteins are implicated in their taxonomic compositions, immunodiagnostic traits, and vaccine development and many of them have been identified as immunomodulators acting at the parasite-host interface [10]. Native ES proteins exposed to the host immune system are the persistent sources of external stimuli that may function as protective antigens to confer naturally acquired immunity [11]. For *H. contortus*, a variety of native ES proteins including AC-5 [12], thiol-binding proteins [13], LDNF glycan antigen [14], and 15- and 24-kDa ES proteins [15] offered partial immune-protective effects to different sheep breeds at varying ages when co-administered with corresponding adjuvants. Simultaneously, a number of the recombinant versions of ES proteins also delivered notable levels of protection against *H. contortus* challenge, such as recombinant HcENO protein [16], recombinant Hcftt-2 protein [17], and recombinant 15- and 24-kDa ES proteins [18].

In our preliminary studies, an ES protein, *H. contortus* α/β-hydrolase domain protein (HcABHD), was ascertained among 114 *H. contortus* ES proteins that interacted with goat T cells via integrated immunoproteomic and bioinformatics [19]. Subsequently, HcABHD was demonstrated as an immunomodulator that impaired host T cell functions via the disruption of T cell viability and proliferation, and the alteration of cytokine production profiles in vitro [20]. As immune factors engaging in the parasite-host interactions might be promising vaccine candidates, HcABHD protein expressed at multiple developmental stages may function as a protective antigen for the development of rational immunoprophylactics targeting *H. contortus*. In the present study, we aimed to conduct vaccine trials to validate the immune protective roles of two HcABHD preparations, recombinant HcABHD (rHcABHD) antigen and anti-rHcABHD IgG, in protecting goats against *H. contortus* infection via two independent trials, and both active and passive immunization achieved the encouraging levels of protection against *H. contortus* challenge.

## Materials and methods

### Ethics statement

All protocols had been reviewed with provincial approval [SYXK (SU) 2010-0005] prior to experiments. All animal studies were carried out to comply with the Guidelines of the Chinese Animal Welfare Council. Daily health conditions of the animals were monitored throughout the experiments.

### Parasite and animals

*Haemonchus contortus* strain (Nanjing strain) was maintained and propagated via serial passages in nematode-free goats in the Laboratory of Veterinary Parasitology, Faculty of Veterinary Medicine, Nanjing Agricultural University, Nanjing, China. The collection of eggs and third-stage larvae (L3) were performed as previously described [21, 22].

Local crossbred and healthy Boer goats (female, 5–6 months of age,) under helminth-free conditions confirmed by fecal egg counts (FEC) were purchased from Prosperous Sheep Inc (Nantong, China) and reared in ventilated cages individually to prevent accidental infections with nematodes in the Animal Experimental Center, Nanjing Agricultural University, Nanjing, China. They were fed hay and whole shelled corn and given access to water in pens ad libitum. Daily observation and physical examination were taken to appraise the health status of each goat throughout the trials.

### Recombinant protein production and generation of polyclonal antibodies (pAbs)

The production of rHcABHD proteins was performed as previously described [20]. Briefly, *Escherichia coli* BL21 (DE3) cells containing the reconstructed pET28a-HcABHD plasmid were incubated with Luria–Bertani medium containing kanamycin (100 µg/mL, Sigma-Aldrich, St. Louis, MO, USA) and then induced by isopropyl-β-d-thiogalactopyranoside (1 mM, Sigma-Aldrich) for rHcABHD expression. The histidine-tag fused rHcABHD protein was obtained from the supernatant of cell lysates (20 mM phosphate, 0.5 M NaCl, 10 mM imidazole) using His-Trap HP purification columns (GE Healthcare, Piscataway, NJ, USA). The rHcABHD proteins were resolved on 12% sodium dodecyl sulfate-polyacrylamide gel electrophoresis (SDS-PAGE) gels for size and purity validation, and the concentration was determined by a bicinchoninic acid (BCA) assay (Thermo Fisher Scientific, Rockford, IL, USA). We employed the Detoxi-Gel Affinity Pak prepacked columns (Thermo Fisher Scientific) to remove lipopolysaccharide contamination in rHcABHD proteins and the purified rHcABHD dissolved in phosphate-buffered saline (PBS) was stored at − 80 °C until further analysis.

To generate antigen-specific pAbs or control goat pAbs, 300 µg of rHcABHD protein or equal volume of PBS blended with Freund complete adjuvant (1:1 in volume; Sigma-Aldrich) was administrated subcutaneously into goats for the primary immunization. Immunized goats were later boosted three times with 300 µg of rHcABHD proteins or equal volume of PBS emulsified in Freund incomplete adjuvant (1:1 in volume; Sigma-Aldrich) after a 2-week interval. One week after the final boost, goat serum containing anti-rHcABHD pAbs or control goat pAbs were harvested from goat peripheral venous blood samples. Specific antibody titer in goat anti-rHcABHD sera was determined as 1:2^21^ by sequential twofold dilution via indirect enzyme-linked immunosorbent assay **(**ELISA), using pre-immunization sera as a negative control as previously described [23]. The purification of serum pAbs was performed by affinity chromatography using Pierce Protein G Agarose (Thermo Fisher Scientific) according to the manufacturer’s protocol, and the concentration of anti-rHcABHD IgG and control goat IgG were determined by the BCA assay (Thermo Fisher Scientific). They were stored at − 80 °C for later use.

### Immunoblot analysis

The rHcABHD protein was resolved on SDS-PAGE gels and transferred onto nitrocellulose membranes. The blots were blocked using 4% bovine serum albumin (BSA; Sigma-Aldrich) in TRIS-buffered saline—0.1% Tween 20 (TBST) for 1 h at room temperature. To check the reactivity and specificity of goat antisera or purified goat IgG, the blots were probed with pre-immunization sera (control) and goat anti-rHcABHD sera (1:500 in TBST), or control goat IgG (control) and anti-rHcABHD IgG (1:2000 in TBST) at 4 °C overnight. Following five washes in TBST, the blots were incubated with horseradish peroxidase (HRP)-coupled rabbit anti-goat (H+L) secondary antibody (Sigma-Aldrich) in TBST (1:5000) for 1 h at 37 °C. After five washes in TBST, the blots were then developed with 3,3′-diaminobenzidine (DAB; Sigma-Aldrich) for 3–5 min and visualized by a ChemiDoc imaging system (Bio-Rad, Hercules, CA, USA).

### Trial 1: experimental design and sampling

**Figure 1:**
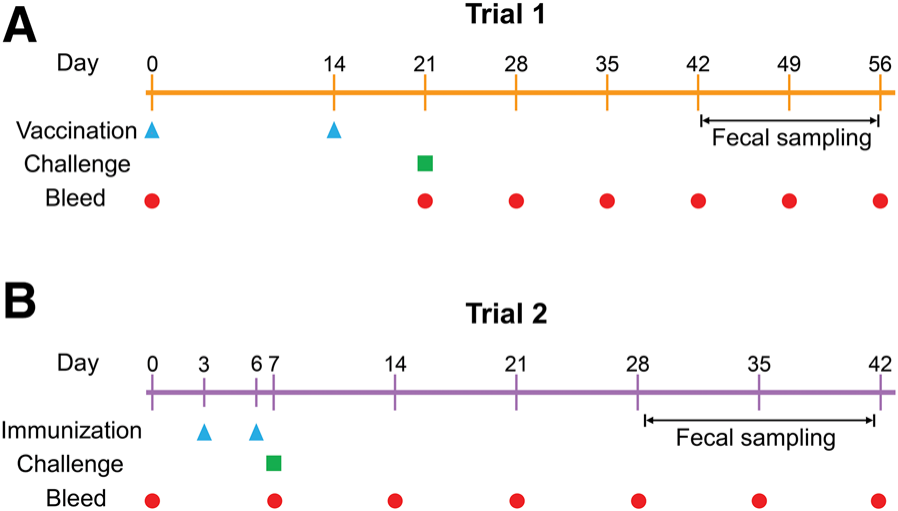
**Experimental protocol scheme.** Goats (n = 5 for each group) were vaccinated/immunized (blue up-pointing triangle), challenged (green square), and bled (red filled circle) at various timepoints during the course of the trials. Fecal samples were collected at the indicated timepoints. **A** Goat experiment timeline of Trial 1 (active vaccination). **B** Goat experiment timeline of Trial 2 (passive immunization).

Prior to the trial, goats were confirmed again to be under helminth-free conditions by FEC, and then randomized and allocated to three groups (n = 5 for each) balanced for weight. The timeline of protocol activities of Trial 1 is indicated in Figure 1A. As rHcABHD protein was dissolved in PBS, goats in the unchallenged adjuvant group (Group A) and challenged adjuvant group (Group C) were administrated subcutaneously with PBS emulsified in Freund complete adjuvant (1:1 in volume; Sigma-Aldrich) at Day 0. A booster immunization with PBS emulsified in Freund incomplete adjuvant (1:1 in volume; Sigma-Aldrich) were given at Day 14. In parallel, goats in the challenged vaccinated group (Group B) were vaccinated with 300 µg of rHcABHD antigen added to Freund complete adjuvant (1:1 in volume; Sigma-Aldrich) and received a booster immunization with 300 µg of rHcABHD antigen plus Freund incomplete adjuvant following the same immunization protocol as Groups A and C. At 1 week post final immunization (Day 21), goats in Groups B and C were orally infected with 5000 infective L3 larvae of *H. contortus* (Nanjing strain) (Figure 1A).

FEC, denoted as eggs per gram (EPG) values, were performed every other day from the initial date of egg excretion (Day 43) until termination based on a modified McMaster method [24]. Cumulative FEC for each goat throughout the study period were evaluated by calculating the area under the curve using the linear trapezoidal method as described elsewhere [25]. All the goats were sacrificed for necropsy on Day 56 of the trial (Figure 1A) and abomasum worm burdens were calculated and enumerated using standard methods as previously described [8]. The reduction of cumulative FEC and abomasal worm burden in vaccinated goats (Group B) was calculated relative to challenged controls (Group C). For the determination of IgA, IgE and total IgG levels in abomasal mucus, abomasal swab samples of each group were gathered post-mortem and processed as described elsewhere [26]. Briefly, abomasal mucus was obtained by gently scraping mucosal surface with sterile glass microscope slides and transferring into 3 mL ice-cold sterile PBS containing 5 mM phenylmethylsulfonyl fluoride (PMSF; Thermo Fisher Scientific) and 5 mM EDTA. The samples were homogenized on the ice for 1 min and centrifuged at 10 000 × *g* for 20 min. The supernatants were collected, divided into aliquots (100 µL), and stored at − 20 °C for later analysis.

Blood samples were obtained from the jugular vein by venipuncture from all goats at various timepoints, namely, before vaccination (Day 0), before challenge (Day 21) and every week after challenge (Day 28, Day 35, Day 42, Day 49 and Day 56) (Figure 1A). Goat serum samples were harvested, collected, aliquoted and stored at − 80 °C for later analysis. Fresh blood samples collected in vacuum blood collection tubes containing K2-EDTA were subjected to BC5000-Vet blood cell analyzer (Mindray, Shenzhen, China) within 1 h for complete blood count (CBC) determination to monitor the health conditions of the goats at each sampling day.

### Trial 2: experimental design and sampling

Goats were randomly assigned to three groups matched for weight: the unchallenged control group (Group D, n = 5), challenged immunized group (Group E, n = 5) and challenged control group (Group F, n = 5). The experimental design of Trial 2 is shown in Figure 1B. Goats in Group E were immunized intravenously with anti-rHcABHD IgG (3 mg) at Day 3 of Trial 2. Subsequently, a booster injection with anti-rHcABHD IgG (3 mg) were administered at Day 6 of the trial. Goats in Groups D and F received an immunization with 3 mg of control goat IgG (obtained in 2.3.) at Day 3 and were given a booster immunization with the same amount of control goat IgG at Day 6. One day after the second immunization (Day 7), goats in Groups E and F were challenged orally with 5000 infective L3 of *H. contortus* (Nanjing strain) (Figure 1B).

The determination of FEC was carried out every 2 days since the primary detection of eggs (Day 29) and cumulative FEC for each goat throughout Trial 2 was estimated (Figure 1B). Goats were euthanized for necropsy on day 35 post challenge (Day 42) and abomasal worm burdens of each group was counted and classified (Figure 1B). The reduction of cumulative FEC and abomasal worm burden in immunized goats (Group E) was evaluated relative to challenged controls (Group F). In addition, abomasal swab samples were collected and managed following the same protocol in 2.4. for IgA, IgE and IgG production determination in abomasal mucus.

Blood samples were taken from all goats before immunization (Day 0), before challenge (Day 7) and every week post infection (Day 14, Day 21, Day 28, Day 35 and Day 42) (Figure 1B). Goat sera samples were harvested, collected, aliquoted and stored at − 80 °C for later analysis of circulating antibody responses, whereas fresh whole blood samples obtained at every sampling day were assayed for CBC analysis.

### ELISA

The specific anti-HcABHD IgG levels in serum of each goat in both Trials 1 and 2 were determined by ELISA assays as previously described [27]. In brief, the indirect ELISA was first developed with the optimal concentration of rHcABHD antigen (100 ng/µL) and the compatible dilution of anti-rHcABHD serum (1:500) by chessboard titration to minimize non-specific background readings. Later, the 96-well plates (Thermo Fisher Scientific) were coated with 100 ng/µL of rHcABHD in carbonate-bicarbonate buffer (50 mM, pH 9.6) at 4 °C overnight. Plates were then washed and blocked with 1% BSA in PBS at room temperature for 1 h. Later, plates were incubated with 100 µL of diluted goat sera (1:500) collected from each group of Trials 1 and 2 at room temperature for 1 h. Following five washes, plates were incubated with HRP-conjugated rabbit anti-goat IgG (H+L) (1:5000, Sigma-Aldrich) at room temperature for 1 h, followed by color development with 3,3′,5,5′-tetramethylbenzidine substrate (Sigma-Aldrich) for 10–15 min. Absorbance at 450 nm (OD450) was determined using a microplate reader (Biotek, Winooski, VT, USA).

The levels of serum and mucosal IgE, IgA, and total IgG were determined using goat IgA, IgE and total IgG ELISA kits (Mlbio, Shanghai, China) according to the manufacturer’s specifications. Serum cytokine levels were detected by goat interleukin (IL)-2, IL-4, IL-10, IL-17A, interferon (IFN)-γ, transforming growth factor (TGF)-β1 and tumor necrosis factor (TNF)-α ELISA Kits (Mlbio) based on the manufacturer’s protocols. One-hundred microliters of serum samples diluted with PBS (1:5) and 100 µL of diluted abomasal swab samples (1:5 in PBS) were applied to ELISA assays, respectively. The limit of quantification dependent upon each analytic kit ranged from between 2 and 800 µg/mL. Each experiment was run in triplicate.

### Statistical analysis

The patterns of FEC data were assessed by fitting generalized additive mixed models (GAMM) in both active and passive immunization trials as described elsewhere [28]. The repeated measures (RM) analysis of variance (ANOVA) methods (based on general linear model) with Bonferroni corrections for multiple comparisons were employed for the statistical analysis of worm burdens using GraphPad Premier 8.0 software (GraphPad Prism, San Diego, CA, USA). Statistical analysis of cumulative FEC was conducted by non-parametric Mann–Whitney tests and the non-parametric Kruskal–Wallis tests were employed for the statistical analysis of abomasal IgE, IgG and IgA levels. Statistical analysis of antigen-specific IgG levels, serum IgE, IgG and IgA levels, serum cytokine secretion levels, and CBC determination were performed by RM-ANOVA with Tukey multiple comparisons test. Differences were regarded as statistically significant when *P*-values were < 0.05. Data were denoted as minimum to maximum (all points) or mean ± standard deviation (SD).

## Results

### Recombinant antigen preparation

The rHcABHD protein was successfully expressed and obtained from the supernatant of cell lysates (Additional file 1A, Lane 1). Following the purification, rHcABHD protein was visualized as a single band with a molecular weight of ~ 36 kDa by Coomassie Blue staining (Additional file 1A, Lane 2). The specificity and reactivity of goat anti-rHcABHD sera or purified anti-rHcABHD IgG was determined by western blot analysis. A single band ~ 36 kDa was observed through the specific recognition of the rHcABHD protein by goat anti-rHcABHD sera (Additional file 1B, Lane 3), whereas no positive band was identified by goat pre-immunization sera (Additional file 1B, Lane 4). Meanwhile, the rHcABHD protein was identified by purified goat anti-rHcABHD IgG as a single band of ~ 36 kDa (Additional file 1B, Lane 5), whereas there was no observed band in the blots probed with control goat IgG (Additional file 1B, Lane 6).

### Parasitological parameter

**Figure 2:**
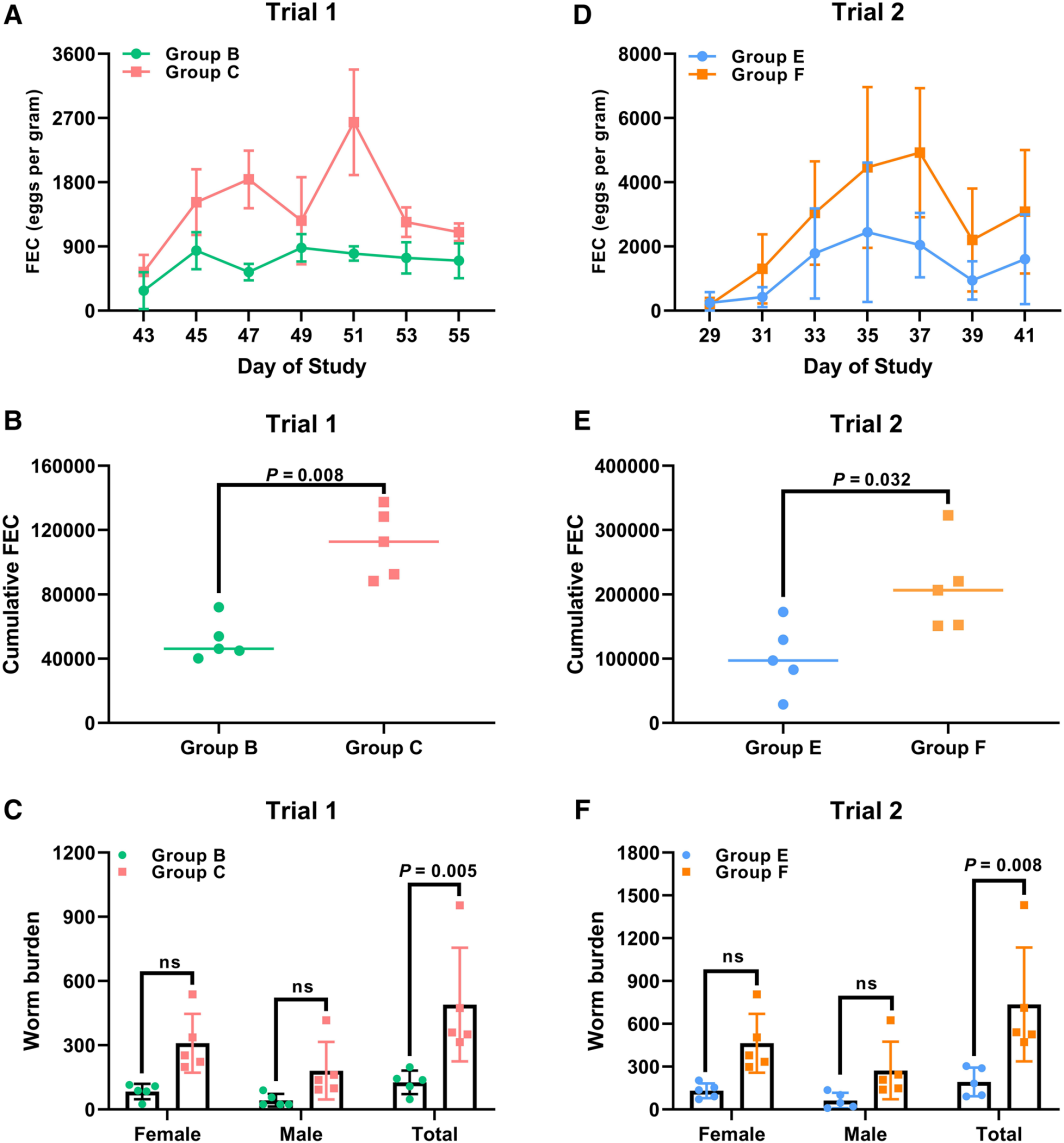
**Parasitological determination of challenged goats in both trials.** **A** Dynamic range of fecal egg counts (FEC) values of challenged goats in Trial 1. Goats in challenged vaccinated group (Group B) and challenged adjuvant group (Group C) started to excrete *H. contortus* eggs in fecal samples around Day 43, and FEC were then performed every 2 days until termination using the modified McMaster method. Eggs per gram (EPG) values were expressed as mean ± SD. Each data point represented the mean FEC value for each group (n = 5). **B** Determination of cumulative FEC for challenged goats in Trial 1. Cumulative FEC values were evaluated by calculating the area under the curve using the linear trapezoidal method. Cumulative FEC values (n = 5 for each group) were presented as minimum to maximum (all points). *P*-values were determined by non-parametric Mann–Whitney tests. **C** Worm burdens of each challenged group in Trial 1. Abomasum worm burdens were differentiated into male, female and total worms. The mean worm burdens (n = 5 for each group) were represented as minimum to maximum (all points), and two groups differed significantly when *P* < 0.05. *ns* not significant. **D** Dynamic range of FEC values of challenged goats in Trial 2. Goats in challenged immunized group (Group E) and challenged control group (Group F) began to excrete *H. contortus* eggs in feces around Day 29, and FEC were then performed every other day until the end of the trial. EPG values were represented as mean ± SD. Each data point represented the mean FEC value for each group (n = 5). **E** Determination of cumulative FEC for challenged goats in Trial 2. Cumulative FEC values were determined by calculating the area under the curve as well. Cumulative FEC values (n = 5 for each group) were presented as minimum to maximum (all points). *P*-values were determined by non-parametric Mann–Whitney tests. **F** Worm burdens of challenged goats in Trial 2. The mean worm burdens (n = 5 for each group) were denoted as minimum to maximum (all points), and two groups differed significantly when *P* < 0.05. *ns* not significant.

Since egg shedding and worm burden are two of the most important efficacy parameters of an anti-*H. contortus* vaccine, we evaluated the dynamic range of FEC values throughout the trials and calculated the reduction rate of abomasal worm burden *postmortem*. In Trial 1 for the active vaccination test, challenged goats in Groups B and C began to excrete *H. contortus* eggs in the fecal samples around Day 43 of the study and the dynamic of FEC values were presented in Figure 2A. The FEC of Group C increased over time and reached the peak levels of 2640 ± 740.3 EPG at Day 51, whereas the peak levels of FEC in Group B reached 880 ± 192.4 EPG at Day 49 (Figure 2A). Overall, GAMM analysis revealed a statistically significant effect of vaccination with rHcABHD antigen on mean FEC over the time-course of Trial 1, showing lower mean FEC in Group B compared to that in Group C (*P* < 0.001). Simultaneously, the vaccination of rHcABHD protein elicited relatively encouraging protection efficacy against *H. contortus*, as exemplified by a 54.0% reduction of cumulative FEC (*P* = 0.008) (Figure 2B) and a 74.2% reduction of total worm burdens (*P* = 0.005) (Figure 2C) for Group B in comparison to Group C (Additional file 2). However, no statistically significant reductions of group mean male (*P* = 0.445) or female (*P* = 0.080) worm burdens were observed in Group B compared to Group C (Figure 2C).

In Trial 2 for the passive immunization test, the excretion of *H. contortus* eggs in the feces of challenged groups was detected since Day 29 of the study and the dynamic range of EPG values were plotted in Figure 2D. The FEC level of Group E reached the peak mean values of 2440 ± 2173 EPG at Day 35, while the highest mean FEC level of Group F was revealed at Day 37, peaking at 4920 ± 2012 EPG (Figure 2D). A statistically significant effect of immunization with anti-rHcABHD IgG on mean FEC over the time-course of Trial 2 was identified by GAMM analysis, with significantly lower mean FEC in Group E than that in Group F (*P* < 0.001). Meanwhile, the immunization of anti-rHcABHD IgG generated a considerable protection against *H. contortus* challenge, as demonstrated by the reductions of cumulative FEC and total worm burdens by 51.5% (*P* = 0.032) (Figure 2E) and 73.8% (*P* = 0.008) (Figure 2F) compared to the challenged control group, respectively (Additional file 2). Analogous to Trial 1, there were no significant differences of female (*P* = 0.121) or male (*P* = 0.519) worm burden between these two challenged groups in Trial 2 (Figure 2F).

### Mucosal antibody responses

**Figure 3:**
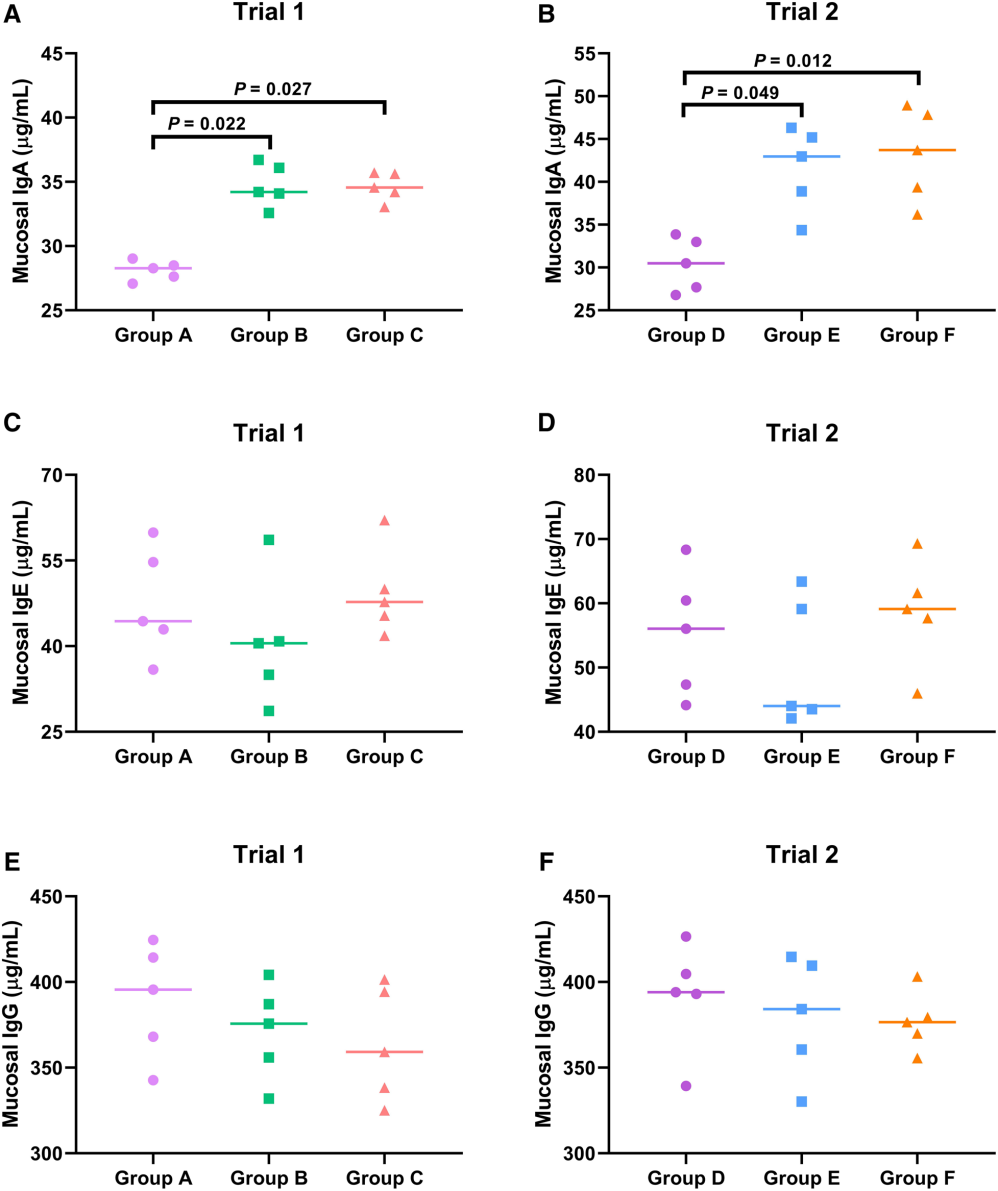
**Determination of abomasal mucosal antibody responses.** Abomasal swab samples were collected at post-mortem from goats in unchallenged adjuvant group (Group A), challenged vaccinated group (Group B) and challenged adjuvant group (Group C), unchallenged control group (Group D), challenged immunized group (Group E) and challenged control group (Group F), and subjected to mucosal IgA, IgE and IgG determinations via ELISA assays. **A** Determination of mucosal IgA levels in Trial 1. **B** Determination of mucosal IgA levels in Trial 2. **C** Determination of mucosal IgE levels in Trial 1. **D** Determination of mucosal IgE levels in Trial 2. **E** Determination of mucosal IgG levels in Trial 1. **F** Determination of mucosal IgG levels in Trial 2. Mucosal antibody levels for each group (n = 5) were represented as minimum to maximum (all points) and non-parametric Kruskal–Wallis multiple comparisons were performed for statistical analysis (*P* < 0.05).

To evaluate mucosal immune responses in the trials, abomasal swab samples were collected and subjected to the determinations of total mucosal IgA, IgE and IgG levels by ELISA assays. In Trial 1, mucosal total IgA levels of challenged goats at necropsy in both Group B (34.7 ± 1.67 µg/mL) and Group C (34.6 ± 1.10 µg/mL) were much elevated with statistical significance (*P* = 0.022 and *P* = 0.027, respectively) compared to the unchallenged goats in Group A (28.1 ± 0.76 µg/mL) (Figure 3A). Consistent to this finding, higher levels of mucosal total IgA productions were detected in challenged goats of Group E (41.5.1 ± 4.92 µg/mL) (*P* = 0.049) and Group F (43.2 ± 5.45 µg/mL) (*P* = 0.012) compared to the unchallenged control group (30.4 ± 3.13 µg/mL) (Figure 3B). However, no significant changes of mucosal total IgA levels between Groups B and C (*P* > 0.05) in Trial 1 or Groups E and F (*P* > 0.05) in Trial 2 were observed (Figure 3A, B). These data suggest that *H. contortus* challenge could induce the amounting production of mucosal parasite-specific IgA, whereas the anti-*H. contortus* preparations of HcABHD did not appear to be efficacious to magnify or alleviate IgA-engaged mucosal immune response. As for the determinations of mucosal total IgE and IgG levels, there were no statistically significant differences of mucosal total IgE or IgG levels among Group A (IgE: 47.6 ± 9.64 µg/mL; IgG: 389.0 ± 33.7 µg/mL), Group B (IgE: 40.7 ± 11.2 µg/mL; IgG: 371.0 ± 28.0 µg/mL) and Group C (IgE: 49.4 ± 7.70 µg/mL; IgG: 363.7 ± 33.6 µg/mL) in Trial 1 (Figure 3C, E). Meanwhile, no observed differences of mucosal total IgE or IgG levels were revealed among Group D (IgE: 55.3 ± 9.80 µg/mL; IgG: 391.6 ± 32.2 µg/mL), Group E (IgE: 50.4 ± 10.0 µg/mL; IgG: 379.8 ± 35.2 µg/mL) and Group F (IgE: 58.7 ± 8.43 µg/mL; IgG: 377.0 ± 17.4 µg/mL) in Trial 2 as well (Figure 3D, F).

### Circulating antibody determination

**Figure 4:**
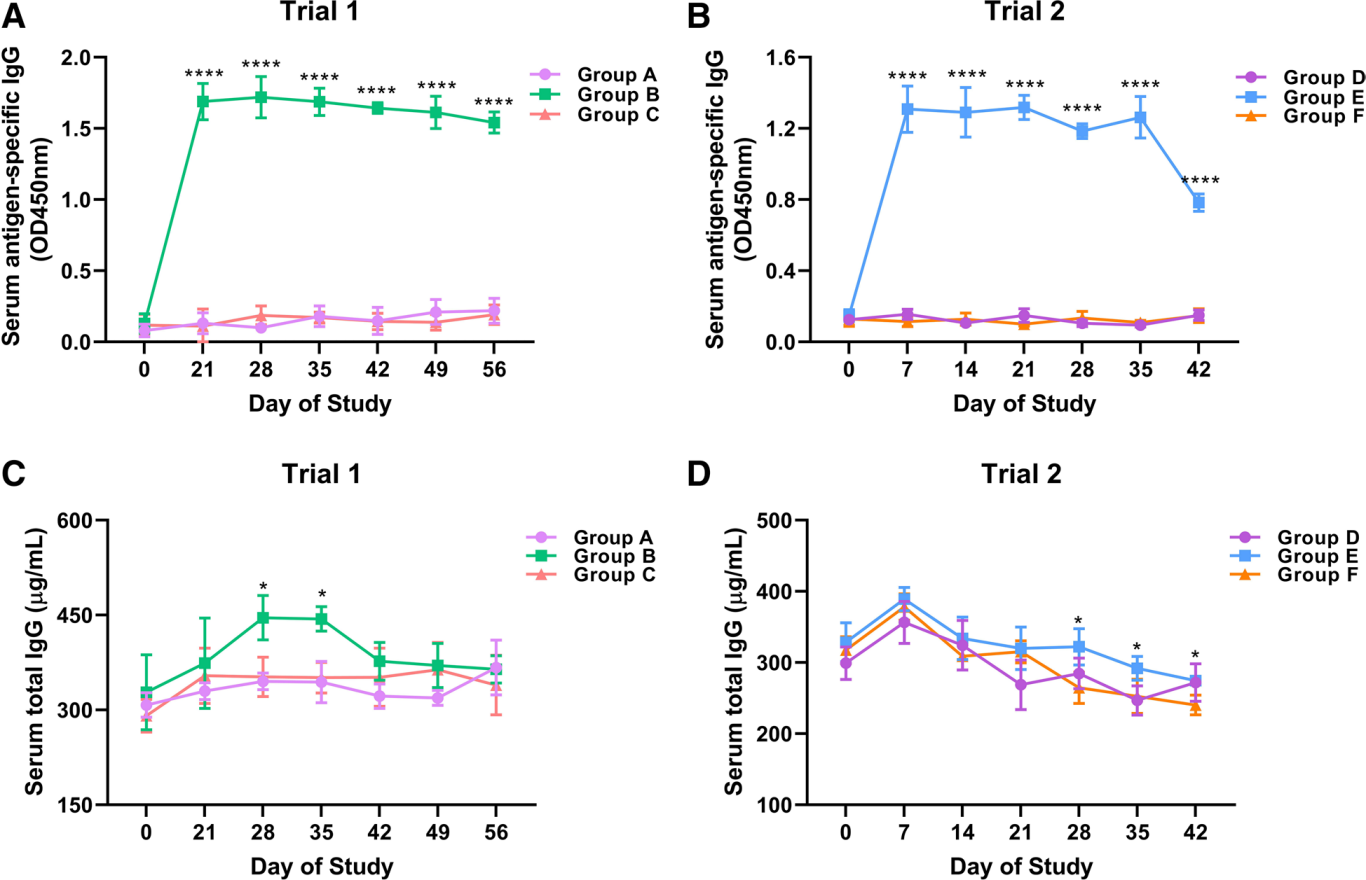
**Dynamics of serum anti-rHcABHD IgG and serum total IgG levels in active and passive immunization trials.** Blood samples were obtained at various timepoints from goats in the unchallenged adjuvant group (Group A), challenged vaccinated group (Group B) and challenged adjuvant group (Group C), unchallenged control group (Group D), challenged immunized group (Group E) and challenged control group (Group F), and serum samples were harvested and assayed for the determination of serum anti-rHcABHD IgG and total IgG levels. **A** Dynamics of serum anti-rHcABHD IgG levels in Trial 1. **B** Dynamics of serum anti-rHcABHD IgG levels in Trial 2. **C** Dynamics of serum total IgG levels in Trial 1. **D** Dynamics of serum total IgG levels in Trial 2. Each data point denoted the mean serum antibody levels (mean ± SD, n = 5 for each group) and asterisks represented statistically significant differences compared to Group C in Trial 1 (*P < 0.05 and ****P < 0.0001) and compared to Group F in Trial 2 (*P < 0.05 and ****P < 0.0001), respectively.

For the assessment of circulating antibody response of unchallenged or challenged goats, serum IgA, IgE and total IgG levels, as well as serum anti-rHcABHD IgG levels, at various timepoints were determined throughout Trials 1 and 2. After the booster immunization with rHcABHD antigen in Trial 1, the levels of anti-rHcABHD IgG in the circulation of goats in Group B spiked beginning on Day 21 of the study (Figure 4A), and serum antigen-specific IgG of vaccinated goats were maintained at relatively higher levels compared to those of adjuvant-immunized goats in Group C (*P* < 0.0001) till the end of the trial (Figure 4A). In Trial 2, serum anti-rHcABHD IgG levels of goats in Group E were elevated following the second passive immunization at Day 7, and remained at considerably higher levels compared to control challenged goats in Group F at all timepoints (*P* < 0.0001) throughout the study (Figure 4B). In addition, the results of ELISA assays revealed that serum total IgG levels of goats in Group B started to augment following the immunization of rHcABHD antigen and reached the peak level at Day 28 (445.9 ± 30.0 µg/mL) (Figure 4C). Comparatively higher levels of serum IgG in Group B were observed than the challenged control group (Group C) at Day 28 (*P* = 0.048) and Day 35 (*P* = 0.015) in Trial 1 (Figure 4C). Concurrently, circulating total IgG levels of immunized goats for Group E remained the highest at Day 7 (389.2 ± 16.5) and then began to decline over the time-course in Trial 2, showing higher serum IgG level at Day 28 (*P* = 0.049), Day 35 (*P* = 0.016) and Day 42 (*P* = 0.011) in comparison to Group F (Figure 4D). In both trials, there were no statistically significant changes of circulating IgA and IgE productions between Groups B and C (Additional file 3A,C), as well as between Groups E and F (Additional file 3B,D) over time. Given that the levels of vaccine-induced antibody are normally associated with vaccine efficacy, the data present here suggest that the protective effects of these two anti-*H. contortus* preparations were mainly mediated by circulating antigen-specific IgG.

### Hematological pathology

**Figure 5:**
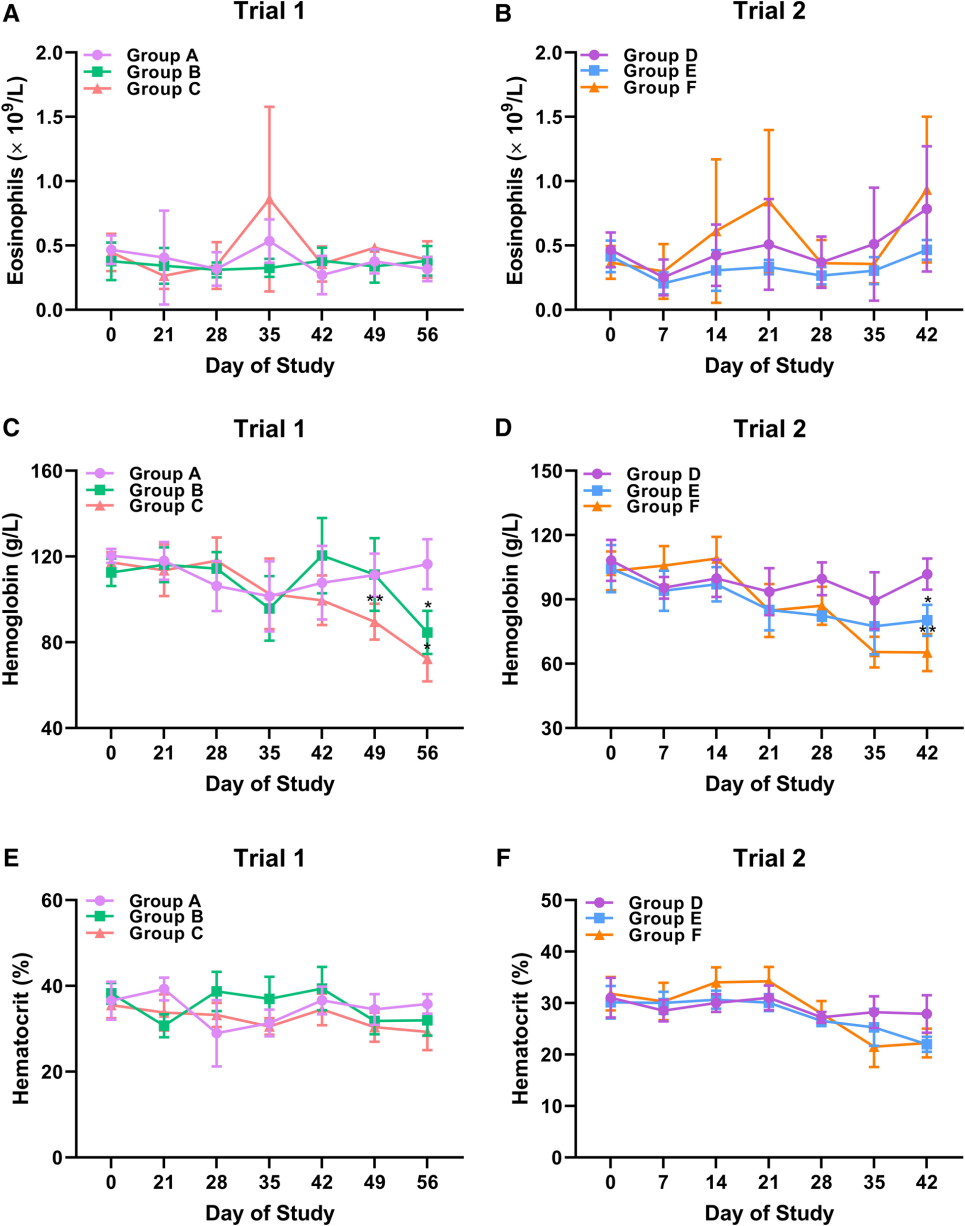
**Blood pathology determination of eosinophil numbers, hemoglobin levels and hematocrit values in active and passive immunization trials.** Fresh blood samples were obtained at each sampling day from goats in unchallenged adjuvant group (Group A), challenged vaccinated group (Group B) and challenged adjuvant group (Group C), unchallenged control group (Group D), challenged immunized group (Group E) and challenged control group (Group F), and subjected to complete blood count (CBC) determination to monitor their health conditions. **A** Dynamics of the numbers of blood eosinophils in Trial 1. **B** Dynamics of the numbers of blood eosinophils in Trial 2. **C** Dynamics of hemoglobin levels in Trial 1. **D** Dynamics of hemoglobin levels in Trial 2. **E** Dynamics of hematocrit values in Trial 1. **F** Dynamics of hematocrit values in Trial 2. Eosinophil numbers, hemoglobin levels and hematocrit values (n = 5 for each group) were represented as mean ± SD, and asterisks represented statistically significant differences compared to Group A in Trial 1 (*P < 0.05 and **P < 0.01) and compared to Group D in Trial 2 (*P < 0.05 and **P < 0.01), respectively.

To monitor the clinical abnormalities of the animals during the trials, fresh blood samples gathered at various timepoints of inoculated goats or mock controls were subjected to blood pathology determination. Although there was an indication that average group eosinophil values increased slightly in Group C at Day 35 and Day 49 of Trial 1, as well as in Group E at Day 14, Day 21 and Day 42 of Trial 2, the elevation of eosinophil numbers was not statistically significant compared to the control groups (*P* > 0.05) (Figure 5A, B). Challenged goats (Groups B, and C) exerted relatively stable hemoglobin levels out to the 3rd week post challenge, whereas lower hemoglobin values were observed in Group B at Day 56 (*P* = 0.031), as well as in Group C at Day 49 (*P* = 0.004) and 56 (*P* = 0.013) of Trial 1 compared to the unchallenged control group (Figure 5C). In Trial 2, significant reductions of hemoglobin levels were observed in Group E (*P* = 0.041) and Group F (*P* = 0.009) at Day 42 compared to the unchallenged controls (Figure 5D). The shift of hemoglobin levels was just revealed near the end of both trials, indicating the correlation of hemoglobin levels with the time-course of *H. contortus* infection. Dynamic of hematocrit values for Trial 1 and Trial 2 were represented in Figure 5E, F respectively. The hematocrit levels of challenged goats (Group B, C, E and F) remained relatively steady and tended to decline at the end of both trials. However, the reduction of hematocrit levels at the 4th and 5th week post challenge was not statistically significant compared to the unchallenged groups (*P* > 0.05) (Figure 5E, F). Simultaneously, no significant changes of white blood cells, neutrophils, lymphocytes, monocytes, basophils, red blood cells in the blood samples were observed between unchallenged and challenged groups in both trials overall (Additional file 4A–L).

### Cytokine production profiles

**Figure 6:**
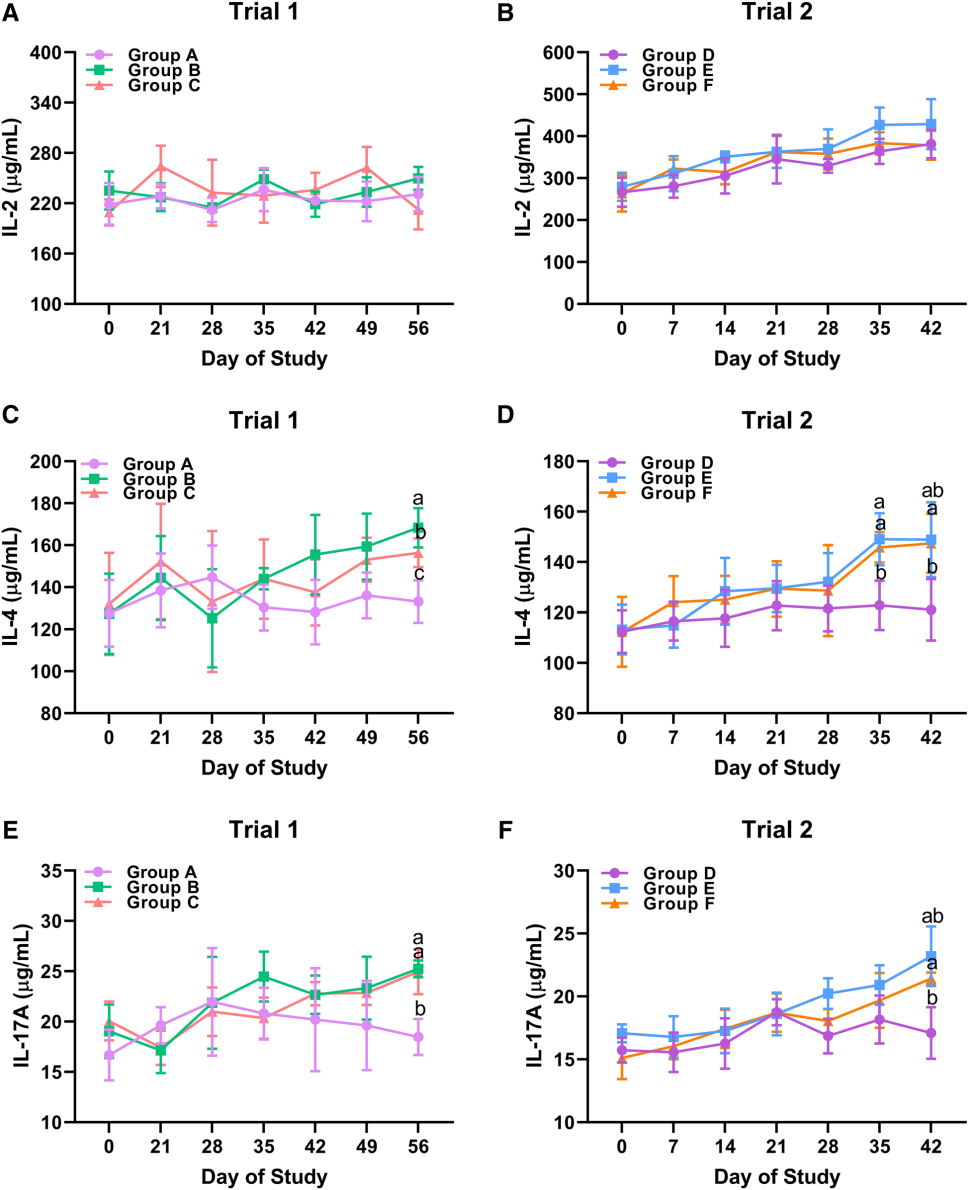
**Circulating cytokine production profiles in unchallenged and challenged goats throughout the trials.** Serum samples obtained at different timepoints from goats in unchallenged adjuvant group (Group A), challenged vaccinated group (Group B), challenged adjuvant group (Group C), unchallenged control group (Group D), challenged immunized group (Group E) and challenged control group (Group F) were subjected to the determination of cytokine production levels. **A** Dynamics of circulating IL-2 levels in Trial 1. **B** Dynamics of circulating IL-2 levels in Trial 2. **C** Dynamics of circulating IL-4 levels in Trial 1. **D** Dynamics of circulating IL-4 levels in Trial 2. **E** Dynamics of circulating IL-17A levels in Trial 1. **F** Dynamics of circulating IL-17A levels in Trial 2. The mean cytokine production levels (n = 5 for each group) were denoted as mean ± SD, and different letters represented significant differences between groups (*P* < 0.05) at the sampling day.

Host protective immunity against *H. contortus* infection depends on the establishment of cellular immune responses associated with a plethora of cytokine productions [4]. Therefore, we next investigated the serum cytokine secretion profiles of goats for all groups throughout the trials. In both trials, IL-2 secretions of unchallenged and challenged goats maintained at a stable level over time, and no significant difference of serum IL-2 levels were observed between unchallenged and challenged groups (Figure 6A, B). As for serum IL-4 productions, challenged goats in both trials exhibited an upward trend of serum IL-4 levels at the end of the study. At Day 56 of Trial 1, goats in Group B (*P* = 0.016) and Group C (*P* = 0.020) exerted higher levels of circulating IL-4 production compared to the unchallenged adjuvant group (Figure 6C). Intriguingly, higher group average IL-4 levels were also observed in Group B in comparison to Group C at Day 56 of Trial 1 (*P* = 0.034) (Figure 6C). At Day 35 of Trial 2, challenged goats (Group E and F) showed higher serum IL-4 levels compared to the unchallenged control group (*P* = 0.034 and *P* = 0.038, respectively) (Figure 6D). Statistically significant elevation of circulating IL-4 levels was observed in Group F (*P* = 0.012) but not in Group E (*P* > 0.05) at Day 42 of Trial 2 (Figure 6D). Circulating IL-17A levels were determined throughout the trials like IL-17 involved in the establishment of tissue repair during parasitic nematode infections [29]. At the 5th week post challenge in both trials, significantly augmented serum IL-17A productions were observed in challenged goats of Group B (*P* = 0.007), Group C (*P* = 0.013) and Group F (*P* = 0.009) compared to the unchallenged controls, but not of Group E (*P* = 0.054) (Figure 6E, F). In addition, no significant differences of serum IL-10, TNF-α, TGF-β1, and IFN-γ secretion profiles were observed between unchallenged and challenged groups over time in both trials (Additional file 5A–H).

## Discussion

Like other parasitic nematodes, the development of promising vaccines against *H. contortus* infections has incorporated the identification of protective immunogenic complexes or antigens like ES immunomodulators and gut-derived antigens [4]. In most cases, vaccinations of these native antigens appear to bring in much higher protective efficacy to *H. contortus* challenge rather than their synthetic or recombinant forms that had compromised or partial protective capacity attributed to lack of post-translational modifications or inaccurate suboptimal folding [30–32]. However, due to the issues of expenditure, biosafety, and quality control with the productions of native *H. contortus* antigens for vaccine commercialization, the exploitation of recombinant versions of novel vaccine candidates that conferred reliable protections to the hosts are still undergoing based on their commercial applicability [4, 33]. In this study, we demonstrate that the recombinant version of HcABHD, an ES immunomodulator acting at the *H. contortus*-host interface in vitro, evoked a substantial protection to *H. contortus*-challenged goats through diminishing cumulative FEC and worm burden by 54.0% and 74.2%, respectively, in an active immunization trial. For a vaccination study, a key factor affecting protective efficacy for the control of *H. contortus* is the level of antigen-specific antibodies (mainly IgG) generated by repeated immunizations, as shown by the protection mechanism of action of Barbervax [34]. Thus, in the parallel passive trial, anti-rHcABHD IgG were employed and elicited a protective capacity by generating a 51.5% reduction of cumulative FEC and a 73.8% reduction of total worm burden. Concurrently, circulating anti-rHcABHD IgG of challenged goats were maintained at considerably high levels throughout both active and passive immunization trials, which might inhibit the development and reproduction of *H. contortus* based on the pivotal role of HcABHD protein in energy metabolism and signaling [20]. As higher transcription level of the HcABHD gene in male adults rather than female adults was determined in our preliminary work [20], a stronger protective pattern of the immunization with rHcABHD antigen or anti-rHcABHD IgG targeting male worms of *H. contortus* was expected. Percentage efficacy (P.E.) of two HcABHD preparations with the reduction in male worm burden were 76.3% and 77.1%, respectively, higher than P.E. for female worm burden (Additional file 2). However, P.E. determined by group mean male or female worm burden was not statistically significant.

Host protective immunity against *H. contortus* is sustained by the establishment of type 2 immune response and the initiation of Th1-type immunity under chronic infections [4, 35]. Protective type 2 immunity is characterized by amounting production of IL-4, IL-13 and IL-5, mediating the impairment of larval development and worm feeding and promoting worm expulsion [35, 36]. In our preliminary work, the external stimuli of rHcABHD protein exhibited modulatory activities on cytokine secretion profiles in vitro and IL-4 secretion of host T cells was significantly inhibited by rHcABHD stimuli [20]. Consistent with this finding, a significantly increased level of IL-4 production was achieved by immunization with rHcABHD protein compared to that in the challenged adjuvant group at Day 42 of Trial 1, which might result from the neutralization of anti-rHcABHD IgG to native HcABHD antigen. Taken together, all these results further validated the immunomodulatory roles of HcABHD on host protective Th2 immunity. Compelling evidence has revealed the augmented IL-4 gene expression in abomasal tissue early after *H. contortus* infection [37, 38], while the observation of elevated serum IL-4 production may vary among early, mid and late infection partly dependent on inoculation dose and animal breeds [39, 40]. As for the late detection of enhanced serum IL-4 production in both trials, it was likely that IL-4 was generated early at the local site of infection and present later in the serum in challenged goats. IL-17 is a regulatory cytokine that engages in inflammatory responses associated with tissue degradation and formation. The initial production of IL-17 promotes rapid tissue repair in response to helminth infections, whereas sustained IL-17 generations could induce tissue inflammation and damage [29, 41]. Initial elevation of serum IL-17A levels were revealed in challenged goats at the 5th week post *H. contortus* challenge in both trials, which might contribute to the early stages of the wound healing process. In addition, host immune protection against *H. contortus* infection is also associated with the elevated numbers of tissue eosinophils and augmented IgA and IgE levels, which engaged in the expulsion of L3, the regulation of L4 feeding, and the induction of hypobiosis [4, 42]. In this study, notable upregulation of mucosal IgA levels, but not serum IgA levels, were observed between unchallenged and challenged groups in both Trials 1 and 2, suggesting the essential role of IgA in host mucosal immunity of *H. contortus*-infected goats. As parasite-specific IgE is mainly involved in the process of rapid rejection of L3 larvae triggered within a short period [43, 44], we did not see any significant difference of mucosal or serum IgE levels between the control group and challenged groups in both trials. Increased tissue eosinophils are observed in both naïve and sensitized sheep during *H. contortus* infections in previous studies [43, 45], and tissue eosinophils are associated with delayed rejection responses against L3 *H. contortus* [4]. In an in vivo study of sheep immunized by repeated infections, tissue eosinophils were shown to be in close proximity to L3 *H. contortus* and were connected with structural damage to tissue larvae [46]. Although the prior study revealed both elevated tissue and blood eosinophils in hyper-sensitized sheep exposed to a secondary infection [47], we herein did not observe increased numbers of blood eosinophils in challenged goats given a primary infection in both Trials 1 and 2. Likewise, there were no observed differences of white blood cells, neutrophils, lymphocytes, monocytes, basophils, and red blood cells between unchallenged and challenged groups in both trials. As chronicity of *H. contortus* infections often results in hematological pathology and associated complications of susceptible animals [48], it was likely that adult worms were present for a too short period to notably affect these hematopathology indexes.

The selection of the adjuvant for a vaccine candidate antigen is of great importance to induce prolonged host protective immunity targeting *H. contortus*. On many occasions, adjuvants have been shown to generate non-specific effects on parasitic nematodes in vaccine trials even in the absence of any nematode-specific antigen component of the vaccine [49, 50]. In prior studies, Freund adjuvants were proved to stimulate high and durable cellular and humoral immune responses when co-administered with recombinant Hco-gal-m/f antigens, whereas no significant protection against *H. contortus* was achieved when Freund adjuvants were applied alone in the vaccination trial [26]. Therefore, to highlight the protective capability of rHcABHD, we employed Freund adjuvants to generate water-in-oil emulsions of immunogens and goats receiving Freund adjuvants alone were set as controls in the active immunization trial. However in the passive immunization experiments, purified control IgG obtained from Freund adjuvants-immunized goats were utilized for the control goats as well. Quil A is a saponin adjuvant adapted for commercial scale production which can activate both Th1 and Th2 cellular immune response and induce humoral responses, whereas DEAE is an aluminum-based adjuvant that can induce a predominant type 2 and antibody-mediated response [51]. As Freund adjuvants cannot be applied to further development of a vaccine for versatile use in the field due to its toxicity potential which may cause tissue damage and painful reactions [52], alternative adjuvants such as Quil A and DEAE might be favorable and merit further investigation.

Alongside the exploitation of novel vaccine candidate antigens associated with immunological parameters like ES proteins, alternative strategies targeting *H. contortus*, such as an integrated immunization regime with multiple antigens that were revealed to confer protective effects, might be promising. The most striking example is the successful immunization against *Teladorsagia circumcincta* with the vaccination regime using eight recombinant antigens, which were designed based on their immunomodulatory potentials [28]. In our preliminary work, a number of *H. contortus* ES antigens identified by immunogenomic and immunoproteomic approaches, including Hc-AK [53], Miro-1 [54], HcSTP-1 [55], HcTTR [56], Hc8 [57] and HcA59 [58], were characterized as immunomodulators acting in the parasite-host interactions via the regulation of the functions of host key effector cells. Instead of employing the conventional vaccine formulation containing single antigen, the immunization strategy administering all the recombinant forms of these antigens in combination, or incorporating other validated candidate antigens like Hc23 [59] and HcENO [16], might confer an augmented level of protection against *H. contortus* and provide an extra option for the development of recombinant subunit vaccines. In summary, we herein validated the protective efficacies of two anti-*H. contortus* preparations of a novel α/β-hydrolase domain protein HcABHD in 5–6 month-old goats via both active and passive immunization trials and achieved a successful outcome. Clearly, future studies are necessary to determine whether rHcABHD could provide encouraging levels of protection to young goats with large sample size because of their susceptibility to *H. contortus*.

## Supplementary information

**Additional file 1.** **Recombinant antigen preparation and western blot analysis.** A: Preparation of rHcABHD antigen. Lane M: standard protein molecular marker; Lane 1: rHcABHD expressed in the supernatant of cell lysates; Lane 2: Coomassie Blue staining of purified rHcABHD protein. B: Validation of the specificity of goat anti-rHcABHD sera and purified anti-rHcABHD IgG by western blot. Lane M: standard protein molecular marker; Lane 3: Immunoblot analysis using goat anti-rHcABHD sera as primary antibody; Lane 4: Immunoblot analysis using goat pre-immunization sera as primary antibody. Lane 5: Immunoblot analysis using purified goat anti-rHcABHD IgG as primary antibody; Lane 6: Immunoblot analysis using control goat IgG as primary antibody.

**Additional file 2.** **The levels of protection of two HcABHD preparations against** ***H. contortus*** **infection.**

**Additional file 3.** **Dynamics of serum IgA and IgE levels in active and passive trials.** Serum samples were harvested from goats in all groups at various timepoints and assayed for the determination of circulating IgA and IgE levels. A: Dynamics of serum IgA levels in Trial 1. B: Dynamics of serum IgA levels in Trial 2. C: Dynamics of serum IgE levels in Trial 1. D: Dynamics of serum IgE levels in Trial 2. The mean serum IgA or IgE levels (n = 5 for each group) were denoted as mean ± SD. In both trials, there were no statistically significant changes of circulating IgA and IgE productions between Groups B and C, as well as between Groups E and F over time.

**Additional file 4.** **Blood pathology analysis of white blood cells, neutrophils, lymphocytes, monocytes, basophils, red blood cells in active and passive trials.** Fresh blood samples were obtained from goats in all groups at each sampling day throughout the trials. A: Dynamics of white blood cell numbers in Trial 1. B: Dynamics of neutrophil numbers in Trial 1. C: Dynamics of lymphocyte numbers in Trial 1. D: Dynamics of monocyte numbers in Trial 1. E: Dynamics of basophil numbers in Trial 1. F: Dynamics of red blood cell numbers in Trial 1. G: Dynamics of white blood cell numbers in Trial 2. H: Dynamics of neutrophil numbers in Trial 2. I: Dynamics of lymphocyte numbers in Trial 2. J: Dynamics of monocyte numbers in Trial 2. K: Dynamics of basophil numbers in Trial 2. L: Dynamics of red blood cell numbers in Trial 2. Each data point represented the mean levels of blood cells for each group (mean ± SD, n = 5), and no significant changes of white blood cells, neutrophils, lymphocytes, monocytes, basophils, red blood cells in the blood samples were observed between unchallenged and challenged groups in both trials overall.

**Additional file 5.** **Circulating IL-10, TNF-α, TGF-β1 and IFN-γ production profiles in unchallenged and challenged goats throughout the trials.** Serum samples obtained at different timepoints from goats in all groups were subjected to the determination of cytokine productions. A: Dynamics of circulating IL-10 levels in Trial 1. B: Dynamics of circulating IFN-γ levels in Trial 1. C: Dynamics of circulating TNF-α levels in Trial 1. D: Dynamics of circulating TGF-β1 levels in Trial 1. E: Dynamics of circulating IL-10 levels in Trial 2. F: Dynamics of circulating IFN-γ levels in Trial 2. G: Dynamics of circulating TNF-α levels in Trial 2. H: Dynamics of circulating TGF-β1 levels in Trial 2. Results were denoted as mean ± SD (n = 5 for each group). No significant differences of serum IL-10, TNF-α, TGF-β1, and IFN-γ secretion levels were observed among all the groups over time.

## Data Availability

The datasets supporting the conclusions of this article are included within Additional file 1, Additional file 2, Additional file 3 and Additional file 4.

## Abbreviations

CBC: complete blood count
ELISA: enzyme-linked immunosorbent assay
EPG: eggs per gram
ES: excretory and secretory proteins
FEC: fecal egg counts
HcABHD: *H. contortus* α/β-Hydrolase domain protein
HRP: horseradish peroxidase
IFN: interferon
IL: interleukin
L3: third-stage larvae
pAbs: polyclonal antibodies
PBS: phosphate-buffered saline
rHcABHD: recombinant HcABHD
TGF: transforming growth factor
TNF: tumor necrosis factor
