# Supplementary figures and images for: Protection studies of an excretory–secretory protein HcABHD against Haemonchus contortus infection
Source: Vet Res. 2021 Jan 6;52:3. doi: 10.1186/s13567-020-00871-0 (PMC7786147; doi:10.1186/s13567-020-00871-0)

**A**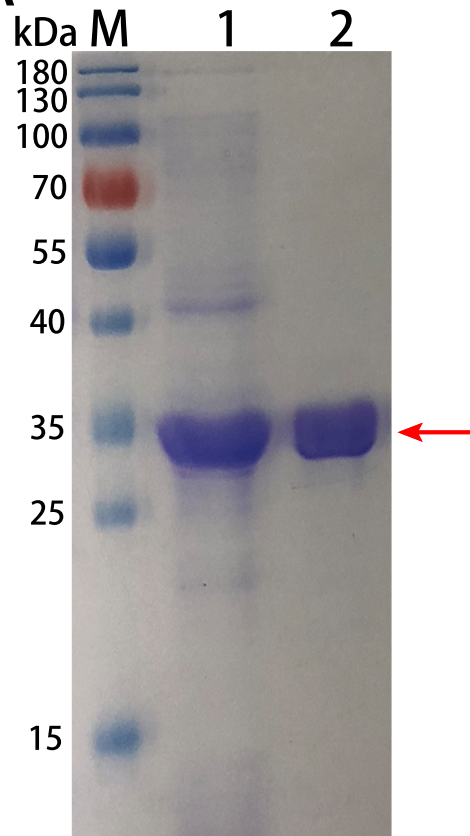**B**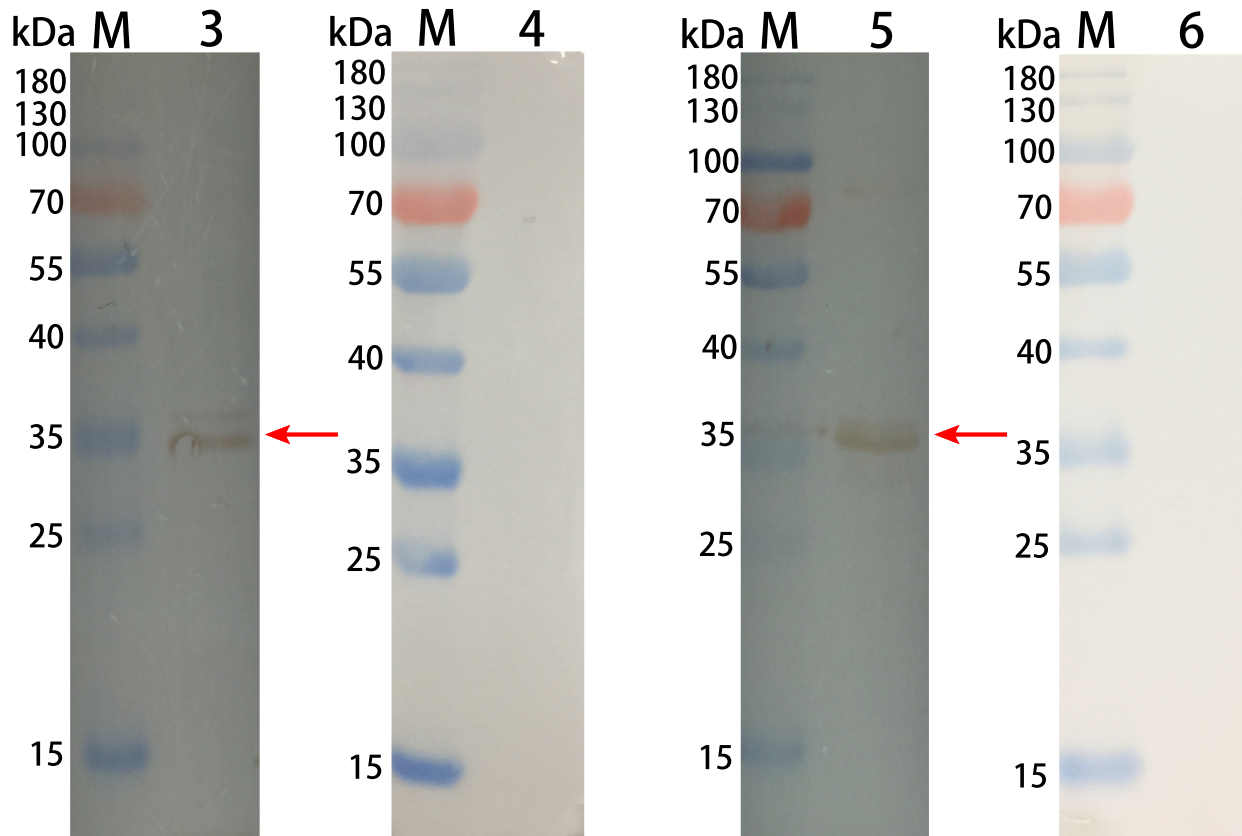

Supplement: Supplementary file 1 — Additional file 1. Recombinant antigen preparation and western blot analysis. A: Preparation of rHcABHD antigen. Lane M: standard protein molecular marker; Lane 1: rHcABHD expressed in the supernatant of cell lysates; Lane 2: Coomassie Blue staining of purified rHcABHD protein. B: Validation of the specificity of goat anti-rHcABHD sera and purified anti-rHcABHD IgG by western blot. Lane M: standard protein molecular marker; Lane 3: Immunoblot analysis using goat anti-rHcABHD sera as primary antibody; Lane 4: Immunoblot analysis using goat pre-immunization sera as primary antibody. Lane 5: Immunoblot analysis using purified goat anti-rHcABHD IgG as primary antibody; Lane 6: Immunoblot analysis using control goat IgG as primary antibody. [file 13567_2020_871_MOESM1_ESM.pdf]

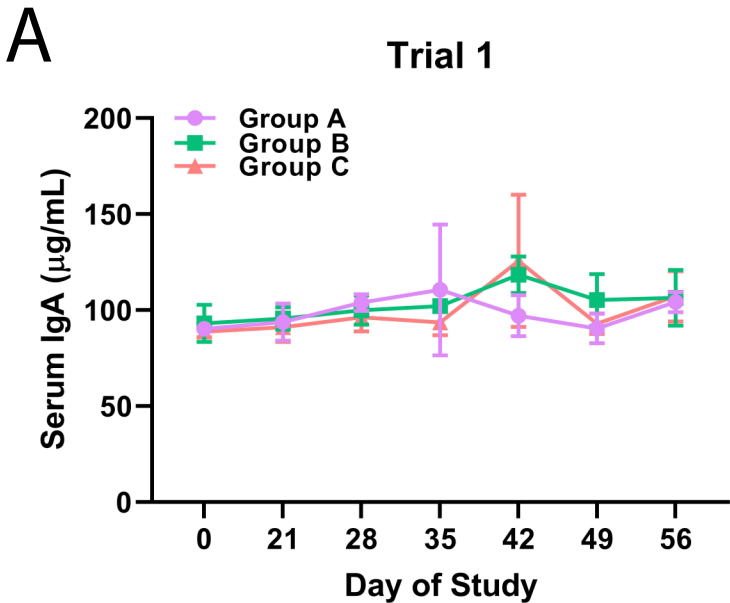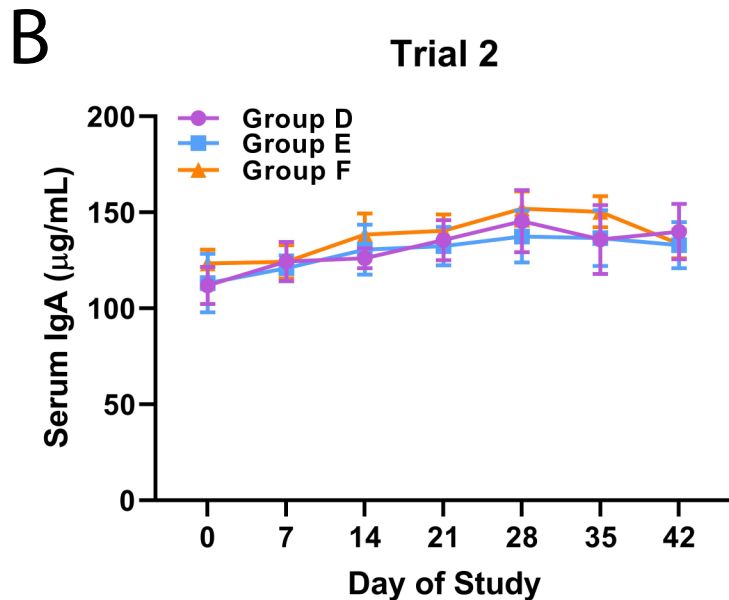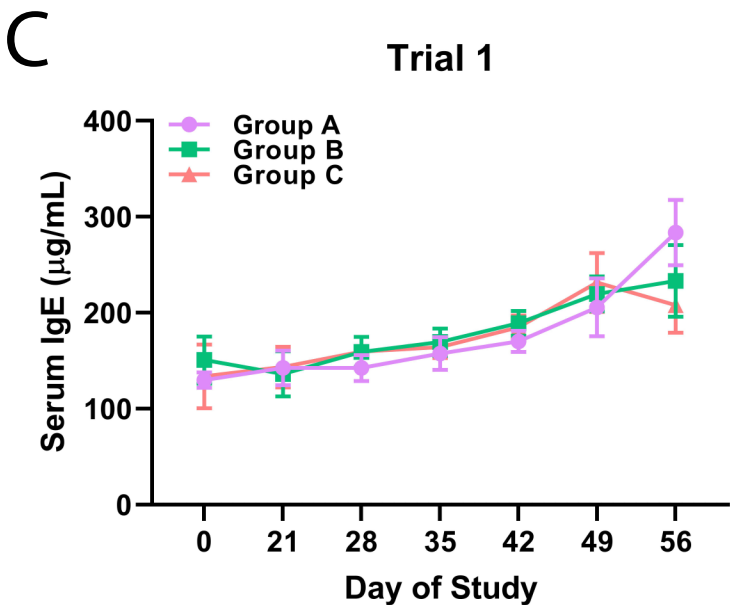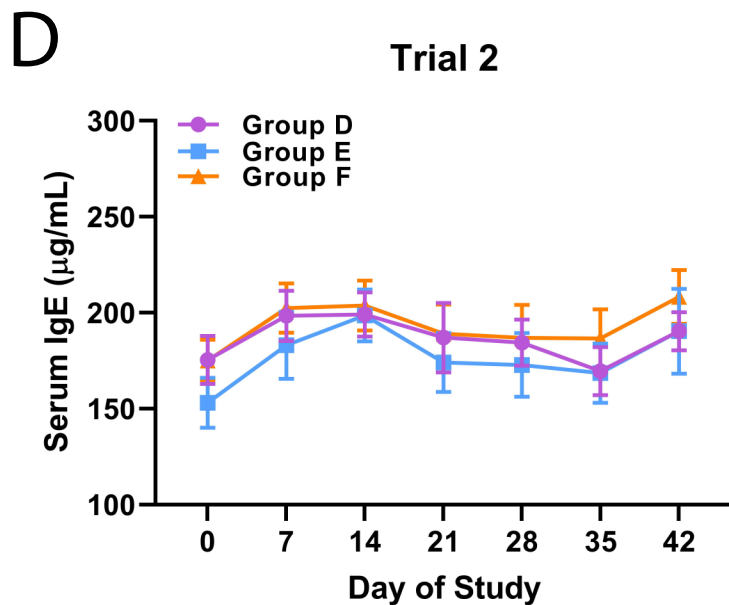

Supplement: Supplementary file 3 — Additional file 3. Dynamics of serum IgA and IgE levels in active and passive trials. Serum samples were harvested from goats in all groups at various timepoints and assayed for the determination of circulating IgA and IgE levels. A: Dynamics of serum IgA levels in Trial 1. B: Dynamics of serum IgA levels in Trial 2. C: Dynamics of serum IgE levels in Trial 1. D: Dynamics of serum IgE levels in Trial 2. The mean serum IgA or IgE levels (n = 5 for each group) were denoted as mean ± SD. In both trials, there were no statistically significant changes of circulating IgA and IgE productions between Groups B and C, as well as between Groups E and F over time. [file 13567_2020_871_MOESM3_ESM.pdf]

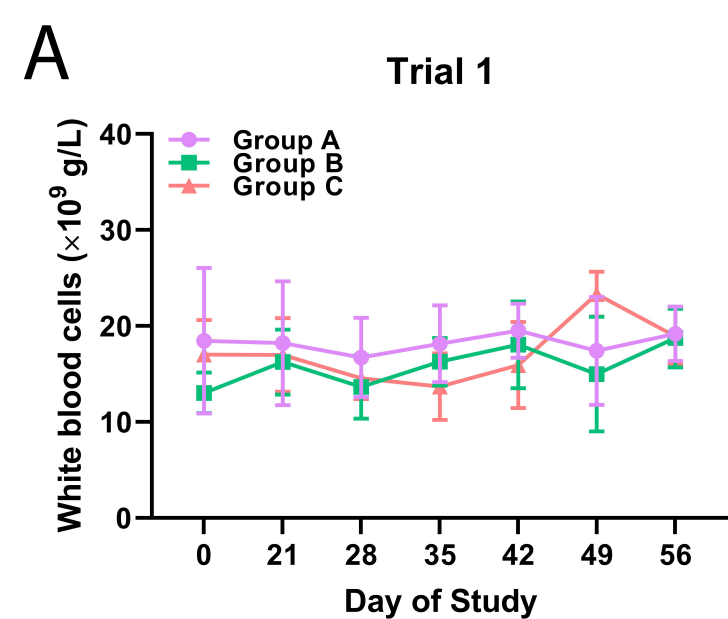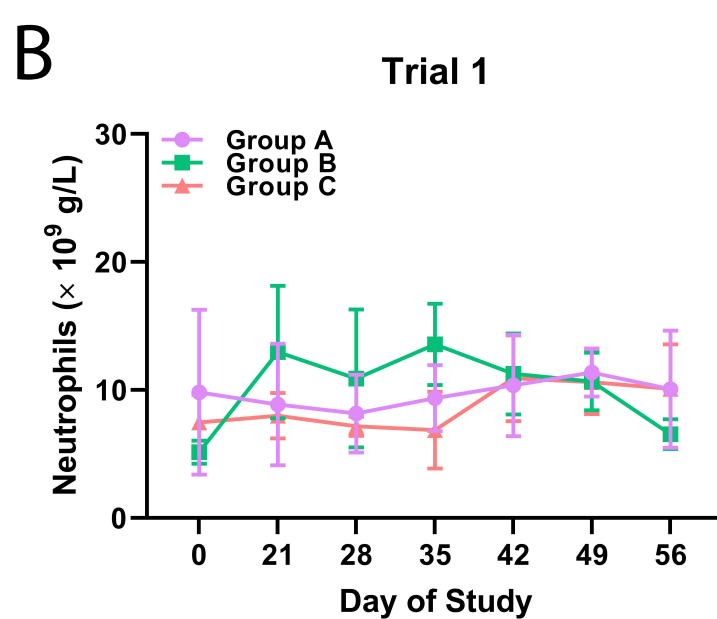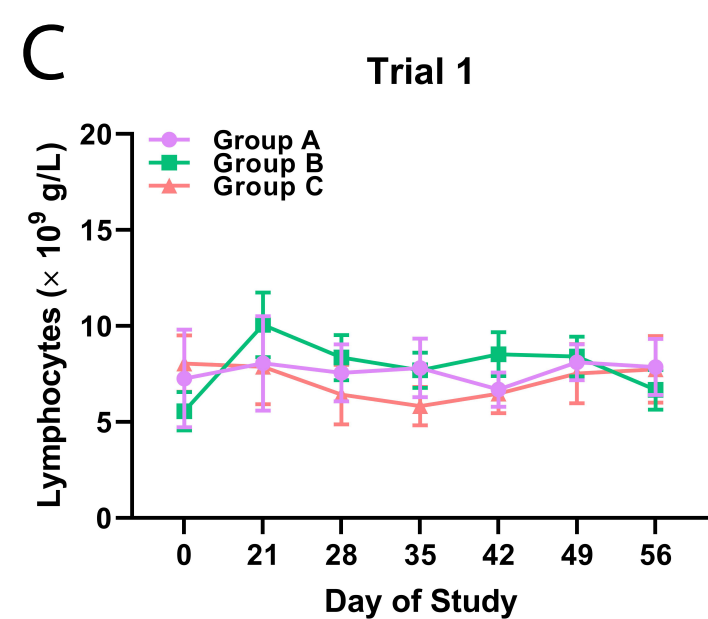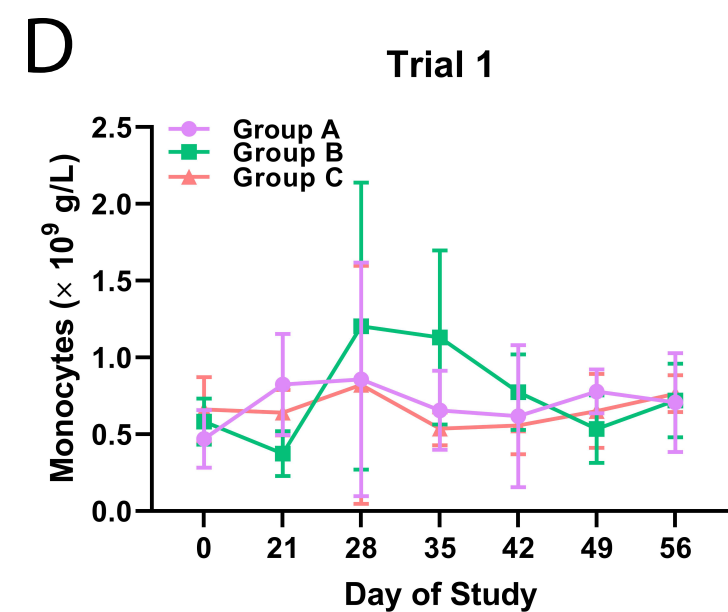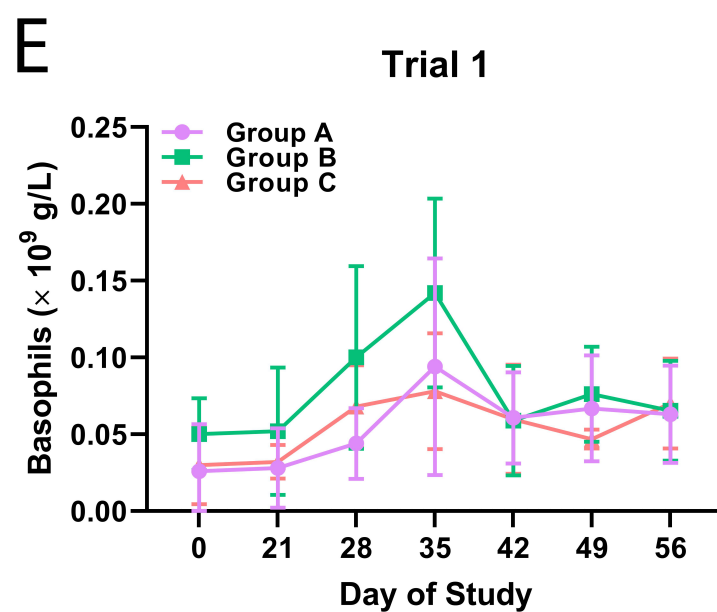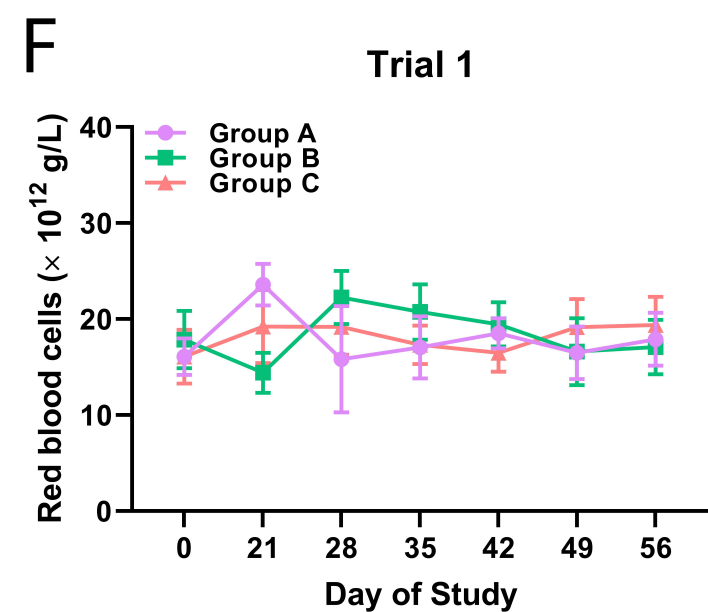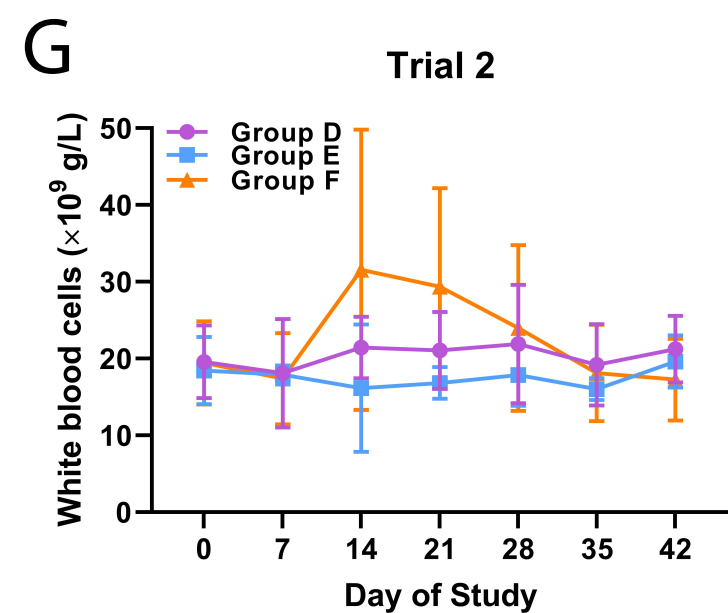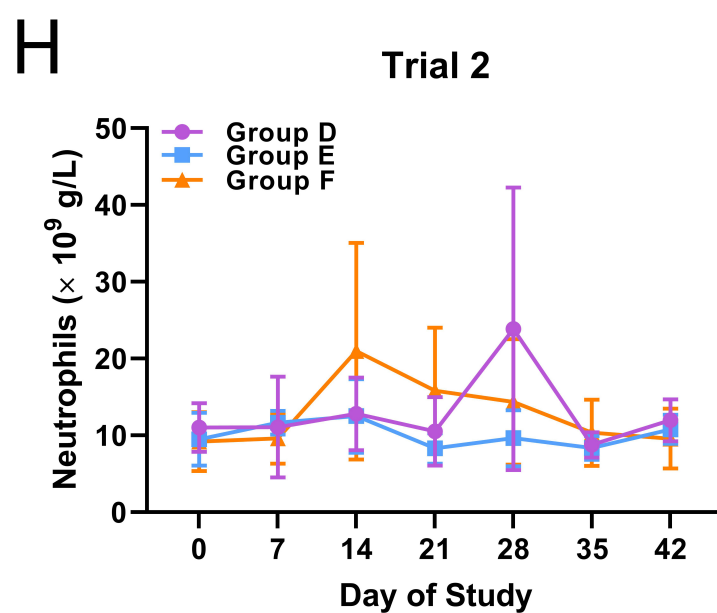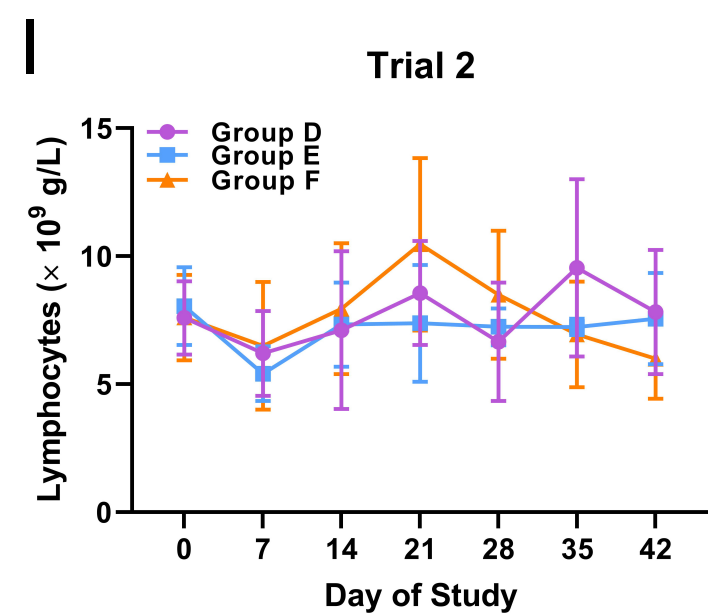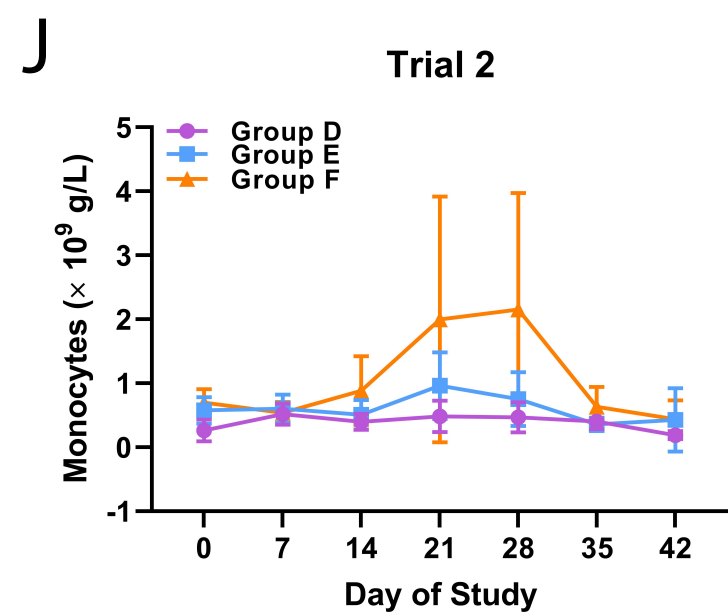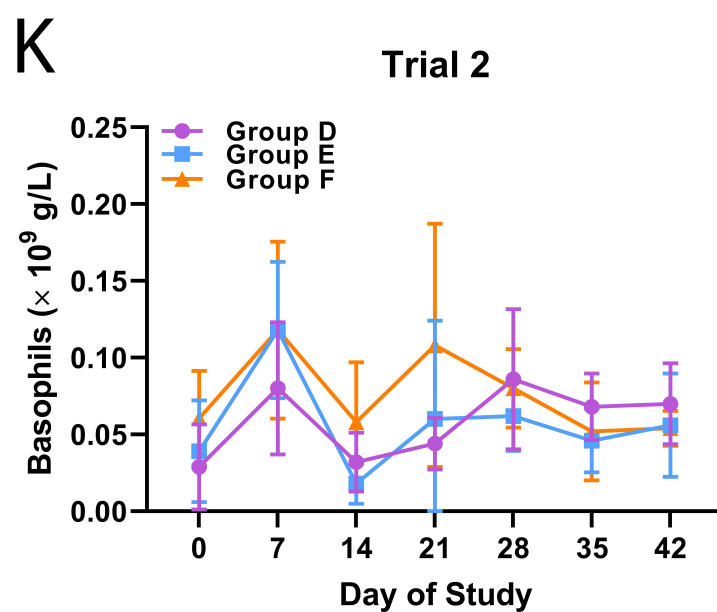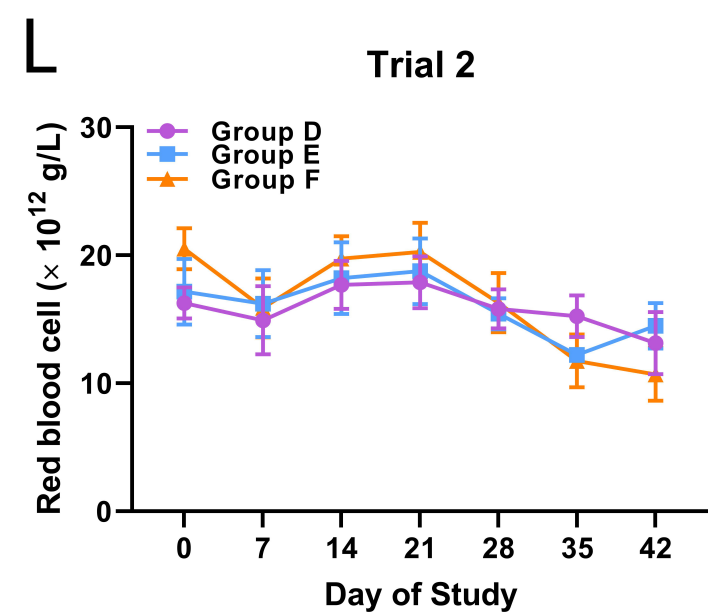

Supplement: Supplementary file 4 — Additional file 4. Blood pathology analysis of white blood cells, neutrophils, lymphocytes, monocytes, basophils, red blood cells in active and passive trials. Fresh blood samples were obtained from goats in all groups at each sampling day throughout the trials. A: Dynamics of white blood cell numbers in Trial 1. B: Dynamics of neutrophil numbers in Trial 1. C: Dynamics of lymphocyte numbers in Trial 1. D: Dynamics of monocyte numbers in Trial 1. E: Dynamics of basophil numbers in Trial 1. F: Dynamics of red blood cell numbers in Trial 1. G: Dynamics of white blood cell numbers in Trial 2. H: Dynamics of neutrophil numbers in Trial 2. I: Dynamics of lymphocyte numbers in Trial 2. J: Dynamics of monocyte numbers in Trial 2. K: Dynamics of basophil numbers in Trial 2. L: Dynamics of red blood cell numbers in Trial 2. Each data point represented the mean levels of blood cells for each group (mean ± SD, n = 5), and no significant changes of white blood cells, neutrophils, lymphocytes, monocytes, basophils, red blood cells in the blood samples were observed between unchallenged and challenged groups in both trials overall. [file 13567_2020_871_MOESM4_ESM.pdf]

**A****Trial 1**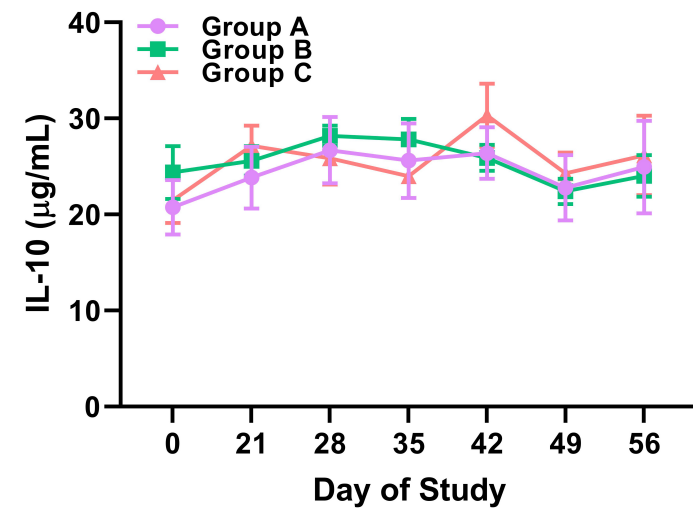**B****Trial 1**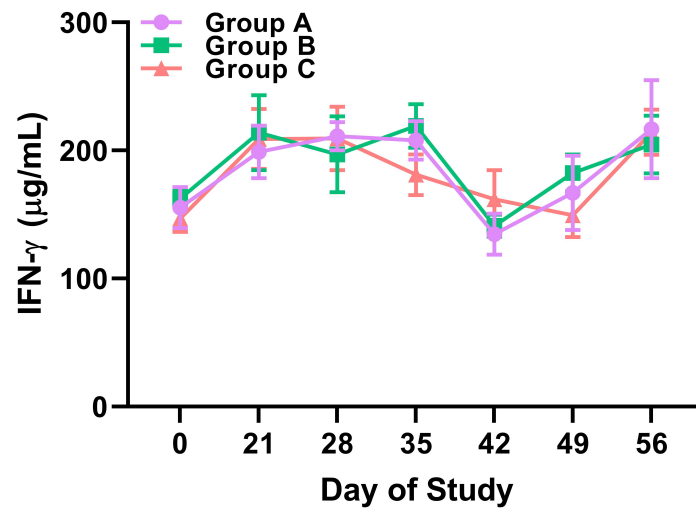**C****Trial 1**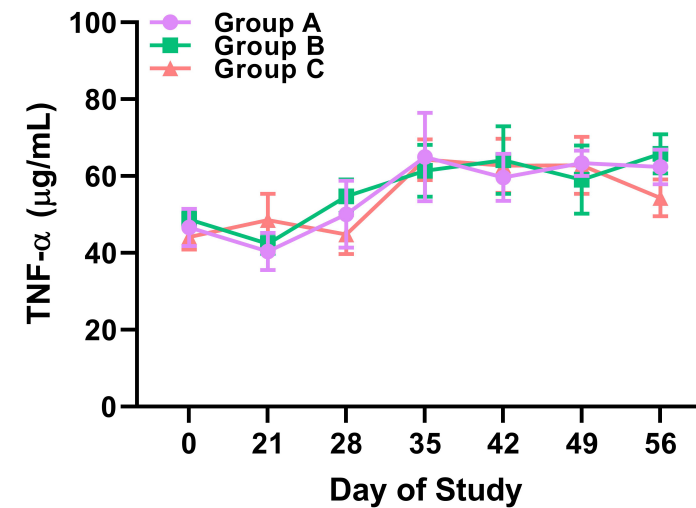**D****Trial 1**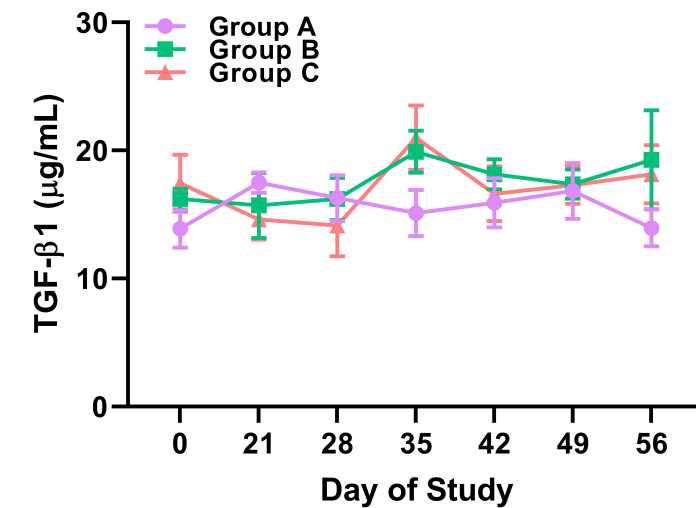**E****Trial 2**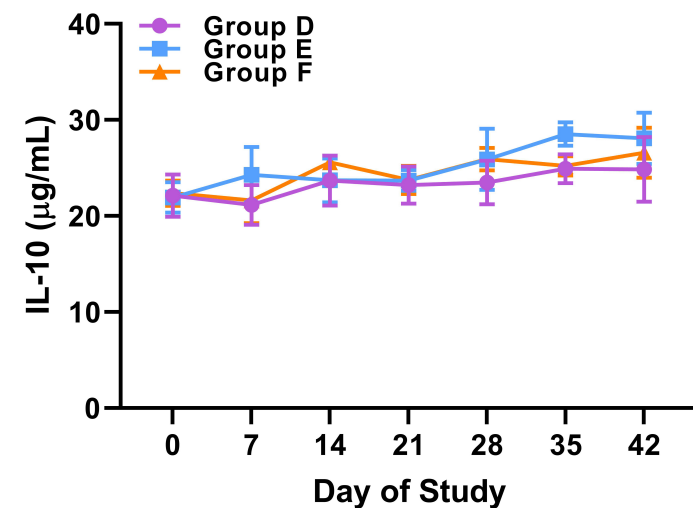**F****Trial 2**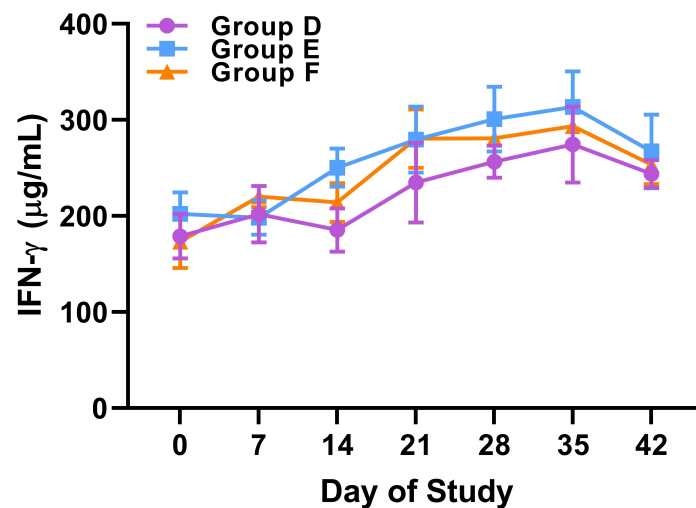**G****Trial 2**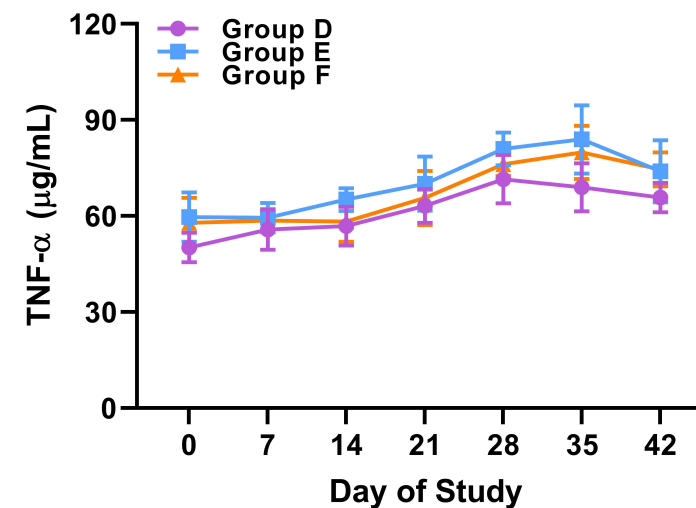**H****Trial 2**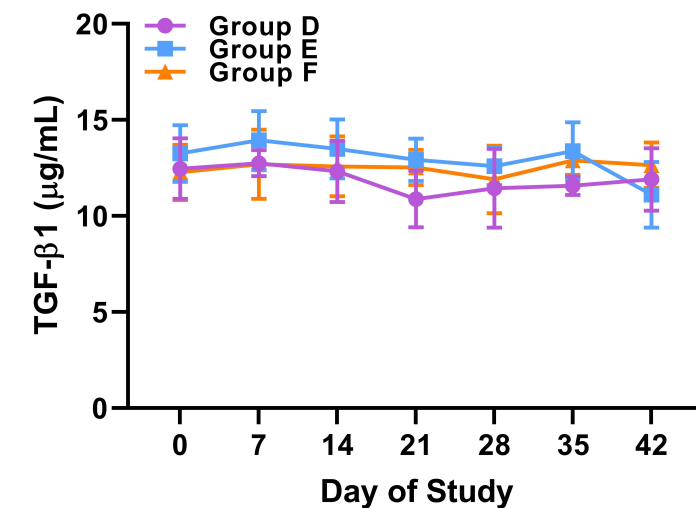

Supplement: Supplementary file 5 — Additional file 5. Circulating IL-10, TNF-α, TGF-β1 and IFN-γ production profiles in unchallenged and challenged goats throughout the trials. Serum samples obtained at different timepoints from goats in all groups were subjected to the determination of cytokine productions. A: Dynamics of circulating IL-10 levels in Trial 1. B: Dynamics of circulating IFN-γ levels in Trial 1. C: Dynamics of circulating TNF-α levels in Trial 1. D: Dynamics of circulating TGF-β1 levels in Trial 1. E: Dynamics of circulating IL-10 levels in Trial 2. F: Dynamics of circulating IFN-γ levels in Trial 2. G: Dynamics of circulating TNF-α levels in Trial 2. H: Dynamics of circulating TGF-β1 levels in Trial 2. Results were denoted as mean ± SD (n = 5 for each group). No significant differences of serum IL-10, TNF-α, TGF-β1, and IFN-γ secretion levels were observed among all the groups over time. [file 13567_2020_871_MOESM5_ESM.pdf]
