# Supplementary material for: Protection studies of an excretory–secretory protein HcABHD against Haemonchus contortus infection
Source: Vet Res. 2021 Jan 6;52:3. doi: 10.1186/s13567-020-00871-0 (PMC7786147; doi:10.1186/s13567-020-00871-0)
Supplement: Supplementary file 2 — Additional file 2. The levels of protection of two HcABHD preparations against H. contortus infection. [file 13567_2020_871_MOESM2_ESM.docx]

**Table S1: The levels of protection of two HcABHD preparations against *H. contortus* infection.**

| **Trial group** | **Egg shedding** | | **Abomasal worm burden** | | | | | |
| --- | --- | --- | --- | --- | --- | --- | --- | --- |
|  | **cumulative FEC** | **P.E.** | **Female worm count** | **P.E.** | **Male worm count** | **P.E.** | **Total worm count** | **P.E.** |
| Group B | 51480 ± 12495 | 54.0%^*^ | 83.40 ± 35.65 | 73.0% | 42.80 ± 29.52 | 76.3% | 126.2 ± 54.89 | 74.2%^*^ |
| Group C | 111840 ± 21594 | NA | 309.0 ± 137.7 | NA | 180.8 ± 134.5 | NA | 489.8 ± 265.7 | NA |
| Group E | 102240 ± 53675 | 51.5%^*^ | 130.6 ± 52.38 | 71.8% | 62.40 ± 53.79 | 77.1% | 193.0 ± 100.6 | 73.8%^*^ |
| Group F | 210600 ± 70025 | NA | 463.6 ± 206.8 | NA | 272.4 ± 201.7 | NA | 736.0 ± 398.7 | NA |

Cumulative faecal egg counts (FEC) values were evaluated by calculating the area under the curve using the linear trapezoidal method and abomasum worm burdens were differentiated into male, female and total worms. Percentage efficacy (P.E.) of vaccinated/immunized goats was calculated relative to challenged controls. Data were presented as mean ± SD and results marked with * were statistically significant compared to challenged controls (*P* < 0.05).
